# Updated protocol of the SANO trial: a stepped-wedge cluster randomised trial comparing surgery with active surveillance after neoadjuvant chemoradiotherapy for oesophageal cancer

**DOI:** 10.1186/s13063-021-05274-w

**Published:** 2021-05-17

**Authors:** Ben M. Eyck, Berend J. van der Wilk, Bo Jan Noordman, Bas P. L. Wijnhoven, Sjoerd M. Lagarde, Henk H. Hartgrink, Peter Paul L. O. Coene, Jan Willem T. Dekker, Michail Doukas, Ate van der Gaast, Joos Heisterkamp, Ewout A. Kouwenhoven, Grard A. P. Nieuwenhuijzen, Jean-Pierre E. N. Pierie, Camiel Rosman, Johanna W. van Sandick, Maurice J. C. van der Sangen, Meindert N. Sosef, Edwin S. van der Zaag, Manon C. W. Spaander, Roelf Valkema, Hester F. Lingsma, Ewout W. Steyerberg, J. Jan B. van Lanschot, on behalf of the SANO-study group

**Affiliations:** 1 Department of Surgery, Erasmus MC Cancer Institute, Erasmus University Medical Centre, Dr. Molewaterplein 40, 3015 GD Rotterdam, the Netherlands; 2 Department of Surgery, Leiden University Medical Centre, Leiden, the Netherlands; 3 Department of Surgery, Maasstad Hospital, Rotterdam, the Netherlands; 4 Department of Surgery, Reinier de Graaf Group, Delft, the Netherlands; 5 Department of Pathology, Erasmus MC – University Medical Centre, Rotterdam, the Netherlands; 6 Department of Medical Oncology, Erasmus MC Cancer Institute, Erasmus University Medical Centre, Rotterdam, the Netherlands; 7 Department of Surgery, Elisabeth Tweesteden Hospital, Tilburg, the Netherlands; 8 Department of Surgery, Zorggroep Twente, Almelo, the Netherlands; 9 Department of Surgery, Catharina Hospital, Eindhoven, the Netherlands; 10 Department of Surgery, Medical Centre Leeuwarden, Leeuwarden, the Netherlands; 11 Department of Surgery, Radboud University Medical Centre, Nijmegen, the Netherlands; 12 Department of Surgery, The Netherlands Cancer Institute - Antoni van Leeuwenhoek Hospital, Amsterdam, the Netherlands; 13 Department of Radiation Oncology, Catharina Hospital, Eindhoven, the Netherlands; 14 Department of Surgery, Zuyderland Medical Centre, Heerlen, the Netherlands; 15 Department of Surgery, Gelre Hospital, Apeldoorn, the Netherlands; 16 Department of Gastroenterology, Erasmus MC – University Medical Centre, Rotterdam, the Netherlands; 17 Department of Radiology and Nuclear Medicine, Erasmus MC – University Medical Centre, Rotterdam, the Netherlands; 18 Department of Public Health, Erasmus MC – University Medical Centre Rotterdam, Rotterdam, the Netherlands; 19 Department of Biomedical Data Sciences, Leiden University Medical Centre, Leiden, the Netherlands

**Keywords:** Oesophageal cancer, Neoadjuvant chemoradiotherapy, Active surveillance, Standard oesophagectomy

## Abstract

**Background:**

The Surgery As Needed for Oesophageal cancer (SANO) trial compares active surveillance with standard oesophagectomy for patients with a clinically complete response (cCR) to neoadjuvant chemoradiotherapy. The last patient with a clinically complete response is expected to be included in May 2021. The purpose of this update is to present all amendments to the SANO trial protocol as approved by the Institutional Research Board (IRB) before accrual is completed.

**Design:**

The SANO trial protocol has been published (10.1186/s12885-018-4034-1). In this ongoing, phase-III, non-inferiority, stepped-wedge, cluster randomised controlled trial, patients with cCR (i.e. after neoadjuvant chemoradiotherapy no evidence of residual disease in two consecutive clinical response evaluations [CREs]) undergo either active surveillance or standard oesophagectomy. In the active surveillance arm, CREs are repeated every 3 months in the first year, every 4 months in the second year, every 6 months in the third year, and yearly in the fourth and fifth year. In this arm, oesophagectomy is offered only to patients in whom locoregional regrowth is highly suspected or proven, without distant metastases. The primary endpoint is overall survival.

**Update:**

Amendments to the study design involve the first cluster in the stepped-wedge design being partially randomised as well and continued accrual of patients at baseline until the predetermined number of patients with cCR is reached. Eligibility criteria have been amended, stating that patients who underwent endoscopic treatment prior to neoadjuvant chemoradiotherapy cannot be included and that patients who have highly suspected residual tumour without histological proof can be included. Amendments to the study procedures include that patients proceed to the second CRE if at the first CRE the outcome of the pathological assessment is uncertain and that patients with a non-passable stenosis at endoscopy are not considered cCR. The sample size was recalculated following new insights on response rates (34% instead of 50%) and survival (expected 2-year overall survival of 75% calculated from the moment of reaching cCR instead of 3-year overall survival of 67% calculated from diagnosis). This reduced the number of required patients with cCR from 264 to 224, but increased the required inclusions from 480 to approximately 740 patients at baseline.

**Conclusion:**

Substantial amendments were made prior to closure of enrolment of the SANO trial. These amendments do not affect the outcomes of the trial compared to the original protocol. The first results are expected late 2023. If active surveillance plus surgery as needed after neoadjuvant chemoradiotherapy for oesophageal cancer leads to non-inferior overall survival compared to standard oesophagectomy, active surveillance can be implemented as a standard of care.

## Introduction

The Surgery As Needed for Oesophageal cancer (SANO) trial is an ongoing phase-III trial that compares active surveillance with standard oesophagectomy for patients with a clinically complete response (cCR; i.e. no evidence of residual disease on diagnostics) to neoadjuvant chemoradiotherapy for oesophageal or oesophagogastric junctional cancer [1]. The trial is designed as a non-inferiority, multi-centre, stepped-wedge, cluster randomised controlled trial. The primary aim is to assess the effectiveness of active surveillance compared to standard oesophagectomy.

Patients are recruited from 12 high-volume centres in the Netherlands. After completion of neoadjuvant chemoradiotherapy (CROSS regimen) [2], two clinical response evaluations (CREs) are performed: the first (CRE-1) at 4–6 weeks and the second (CRE-2) at 10–14 weeks after completion of neoadjuvant chemoradiotherapy. CRE-1 consists of endoscopy with bite-on-bite biopsies and CRE-2 consists of 18F-FDG PET/CT, followed by endoscopy with bite-on-bite biopsies and endoscopic ultrasonography (EUS) with fine-needle aspiration of suspected lymph nodes. If a patient has cCR at CRE-2, the patient will be assigned to either standard oesophagectomy or active surveillance, depending on which study arm the participating hospital was recruiting for (according to the stepped-wedge, cluster randomised design) [1]. If locoregional residual or distant disease is detected during one of these CREs, the patient is excluded from further follow-up within the study.

**Fig. 1:**
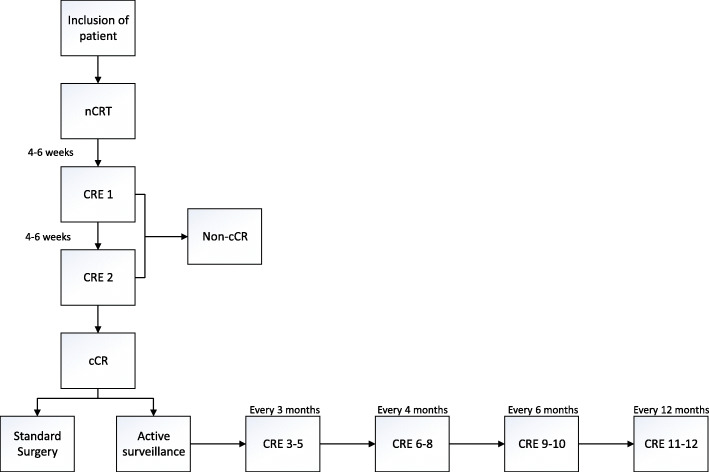
Schematic overview of the SANO trial, comparing active surveillance with standard oesophagectomy in patients with oesophageal cancer and a clinically complete response after neoadjuvant chemoradiotherapy. Patients in whom no residual tumour is detected at two clinical response evaluations after neoadjuvant chemoradiotherapy are considered to have a clinically complete response. Patients who do not have a clinically complete response will undergo oesophagectomy in case no distant metastases are detected. If patients have residual disease at one of the clinical response evaluations during active surveillance (CRE 3–12), postponed oesophagectomy will be performed in case no distant metastases are detected and active surveillance will be stopped. nCRT neoadjuvant chemoradiotherapy, CRE clinical response evaluation, cCR clinically complete responder

Patients in the active surveillance arm undergo diagnostic evaluations similar to CRE-2 every 3 months in the first year, every 4 months in the second year, every 6 months in the third year, and yearly in the fourth and fifth year. During active surveillance, oesophagectomy will be offered only to patients in whom locoregional regrowth is highly suspected or proven in the absence of distant metastasis. A schematic overview of the SANO trial is provided in Fig. 1.

Ethical approval for the study has been obtained from the institutional review board (IRB) of the Erasmus MC (MEC2017–392). The trial has been registered in the Netherlands Trial Register (NTR 6803) and is being conducted in accordance with the Declaration of Helsinki (10th version, Fortaleza, 2013) and the Dutch Medical Research Involving Human Subjects Act (WMO).

The original SANO trial protocol has been published in *BMC Cancer* in 2018 (10.1186/s12885-018-4034-1) [1]. Patient accrual has been started in November 2017, and the last patient with a clinically complete response is expected to be included in May 2021. Following the publication of the protocol and start of the trial, amendments have been made to the protocol to reflect new insights about the accuracy of the CREs and survival of the study population and to further clarify the protocol regarding study procedures. The amendments to the protocol have been approved by the IRB of the Erasmus MC. The purpose of this update is to present all amendments to the SANO trial protocol before accrual will be completed.

## Study design

Two amendments have been made to the study design.

According to the stepped-wedge cluster randomised trial design, clusters of centres are randomised from the control arm to the experimental study arm. The initial trial protocol stated that the centres in the first cluster would not be randomly determined, but would consist of Erasmus Medical Centre (coordinating centre and sponsor of the trial) and Zuyderland Medical Centre. Both centres have extensive experience in performing CREs and included a large number of patients in the preSANO trial, ensuring maximal safety for introduction of the novel active surveillance strategy [3]. Meanwhile, there was another centre that gained extensive experience within the preSANO trial. To provide a random effect to this first cluster but ensure optimal patient safety, we randomly assigned either Zuyderland Medical Centre or Catharina Hospital to the first cluster together with the Erasmus MC.

Since the cCR rate and the rate of crossover is variable (see the “Statistical analysis” section), it is not possible to determine an exact number of patients that need to be included at baseline to end up with exactly the correct number of patients with cCR. Therefore, to ensure that we do not end up with a sample size that is too small and thus an underpowered trial, we will continue including patients at baseline until we reach the predetermined number of patients with cCR. As a result, some patients will be included at baseline but will not have reached the moment of cCR yet, while the baseline enrolment of the trial will be stopped. These additional patients will be included in the analysis of the trial to increase the statistical power.

## Study population

Three amendments have been made to the eligibility criteria.

First, a new exclusion criterion has been added to the protocol to exclude patients who have had diagnostic or therapeutic endoscopic treatment (e.g. endoscopic mucosal resection or endoscopic submucosal dissection) before the start of neoadjuvant chemoradiotherapy. According to the eligibility criteria of the initial protocol, these patients could have been included in the trial at this moment. However, since the oesophageal tumour, and especially the luminal side of the tumour, has been largely removed by the endoscopic resection, accurate detection of locoregional residual disease by means of endoscopic bite-on-bite biopsies and follow-up with PET/CT might be hampered. These patients are probably at increased risk of having undetected residual disease during the CREs and are thus possibly at increased risk of developing a non-resectable regrowth.

Second, a small number of patients could not decide to participate in the SANO trial before chemoradiotherapy was started. Since the first CRE is not planned until 4–6 weeks after completion of chemoradiotherapy, the trial protocol was amended to allow patients to be included during or shortly after completion of chemoradiotherapy. This might result in some missing baseline health-related quality of life questionnaires.

Third, the initial trial protocol dictated that patients with histologically proven squamous cell carcinoma or adenocarcinoma are eligible. An amendment was made that whenever pathology is inconclusive but a multidisciplinary tumour board concludes that there are sufficient (clinical) arguments for the diagnosis of oesophageal carcinoma (e.g. because of a radiologically, endoscopically, and/or endosonographically highly suspected lesion) and subsequent treatment is neoadjuvant chemoradiotherapy followed by surgery, patients are eligible for the study as well. This situation occurs, however, very rarely. An example of such a situation is as follows: a patient who is known with a history of Barrett’s oesophagus presents with increasing dysphagia and weight loss. Endoscopy shows a tumorous lesion within the Barrett segment in the distal oesophagus, of which biopsies are taken. Endoscopic ultrasonography shows a cT3 tumour without positive lymph nodes. The PET/CT scan shows an intense FDG-avid lesion in the distal oesophagus without positive lymph nodes and no distant metastases. The diagnostic CT scan also shows a distal oesophageal tumour without nodal and distant metastases. Eventually, pathology of the biopsies shows high-grade dysplasia, with suspicion of but unconfirmed invasive carcinoma. Despite the absence of confirmation of invasive carcinoma, the patient is enrolled in the SANO trial and neoadjuvant chemoradiotherapy is started.

## Study algorithm

Four amendments have been made to the study algorithm.

First, the targeting of endoscopic biopsies can be hindered and the pathological assessment of residual tumour cells in the biopsy specimen at CRE-1 can be unreliable due to radiation effects and inflammation. To avoid a high rate of false positives at CRE-1 and since it is known that surgery can be safely postponed up to 10–14 weeks after completion of chemoradiotherapy, patients proceed to CRE-2 if the outcome of the pathological assessment is uncertain at CRE-1 [4, 5]. Of note, if patients have uncertain outcome of the pathological assessment of the biopsy specimen at CRE-2, patients will not be allowed to continue in the trial and will undergo surgery, as information on the safety of further postponement of surgery is lacking.

Second, the initial trial protocol described that at CRE-2, patients with (cyto) histological evidence of locoregional residual disease or highly suspected locoregional residual disease on PET/CT without distant metastases will undergo surgery, whereas patients without (cyto) histological evidence of residual disease are considered cCR. We clarified the protocol and stated that patients who have suspected lymph nodes on EUS which are unreachable with fine-needle aspiration are not considered cCR. If in the short term no representative cytology can be obtained from suspected lymph nodes during CRE-2, the patient will also not be considered cCR.

Third, the accuracy of endoscopic bite-on-bite biopsies is compromised if a smaller biopsy instrument is being used, for instance a paediatric endoscope. For this reason, we amended the protocol to state that patients who have a stenosis which cannot be passed with a normal Q-endoscope during endoscopy at CRE-1 or CRE-2 will not be considered cCR, regardless of traversability with the paediatric endoscope. Comparably, in patients who have a stenosis that cannot be passed with the ultrasound endoscope during CRE-II, an ultrasonographic assessment of lymph nodes cannot be performed beyond the stenosis, compromising complete assessment of the regional lymph nodes. Therefore, patients with a non-passable stenosis during EUS will not be considered cCR.

Fourth, the initial trial protocol dictated that CRE and surveillance biopsies with uncertain outcome or with high-grade dysplasia would have to be revised at the Department of Pathology of the Erasmus MC. However, often this is not logistically feasible, as for safety reasons patients have to undergo surgery as soon as possible after a positive biopsy. Therefore, the amendment states that biopsies can be revised by a second independent expert GI pathologist in the participating centre following the same strategy, using a standard protocol. In case of discordant results, the specimens will be reviewed by a third independent expert GI pathologist and a consensus diagnosis should be reached if at least two pathologists agree. In case the revision concludes high-grade dysplasia, the CRE will be considered positive. In case the results remain uncertain, a multidisciplinary tumour board at the Erasmus MC will reach consensus on further treatment, taking into account the condition of the patient and other diagnostic modalities such as 18-FDG PET-CT.

## Follow-up

One amendment has been made to the follow-up of patients.

To compare distant dissemination between both treatment arms, the initial trial protocol described that patients included in the standard surgery arm have to undergo PET-CT scans at 12 and 24 months after neoadjuvant chemoradiotherapy. However, the rationale for the timing of these scans was a 12- and 24-month follow-up period after surgery in the standard surgery arm, which translates to a longer follow-up period when calculated from completion of neoadjuvant chemoradiotherapy. To reach sufficient follow-up time for the development of metastases and thus make a fairer comparison between the two study arms, the timing at which the follow-up PET/CT scans are planned in the standard surgery arm has been changed from 12 and 24 months to 16 and 30 months after neoadjuvant chemoradiotherapy. These points in time match the sixth and ninth clinical response evaluations (CRE-6 and CRE-9) in the active surveillance arm at which PET/CT scans are also made. In this way, a distant dissemination rate can be calculated at these exact points in time.

## Study parameters/endpoints

No amendments have been made to the study parameters/endpoints.

## Safety and stopping rules

No amendments have been made to the safety and stopping rules.

## Statistical analysis

One amendment has been made to the statistical analysis.

Initially, it was calculated that 264 patients with cCR would be required to demonstrate that active surveillance is non-inferior to standard surgery. For this sample size calculation, an expected 3-year overall survival of 67%, non-inferiority margin of 15%, intra-centre correlation coefficient of 0.02, power of 80%, and significance level of 0.05 were used. These survival data were based on the CROSS trial and defined from the moment of randomisation (i.e. pre-treatment) [2, 6]. Based on preliminary data of the preSANO trial, the initial sample size calculation accounted for a 50% cCR rate and a 12% drop-out rate (e.g. patients with cCR within the surgery arm who request active surveillance arm, or vice versa; so-called crossover patients). Moreover, to reduce the number of newly included patients needed and to optimally use the data from the preSANO trial, 60 patients with cCR from the preSANO trial were expected to be included in the SANO trial.

Based on the final data of the preSANO trial and monitoring data of the first part of the SANO trial, it appeared that the cCR rate was 34% and the crossover percentage was 20%. Moreover, it appeared that only 29 instead of 60 patients with cCR from the preSANO trial met all criteria of the SANO trial and could be included. Also, new data on survival of patients with cCR after neoadjuvant chemoradiotherapy have become available [7]. Based on these data, the sample size has been recalculated. The sample size was recalculated with expected 2-year overall survival of 75%, defined from the moment at which patients reach cCR (which is approximately 5 months after diagnosis). Accordingly, the sample size was recalculated with the predetermined power of 80%, significance level of 0.05, non-inferiority margin of 15%, and intra-centre correlation coefficient of 0.02. As a result, 224 patients (i.e. 112 patients in each arm) with cCR will have to be enrolled in the trial. With a crossover rate of 20%, the total number of required inclusions will be 280 (= 224/0.8) patients with cCR. Taking into account that 29 patients with cCR can be included from the preSANO trial and a cCR rate of 34%, this will translate into approximately 740 patients required at baseline.

Simulating trial outcomes on 2-year overall survival calculated from the moment of cCR (approximately 5 months after diagnosis) is justified compared to 3-year overall survival calculated from diagnosis, as the power and significance levels are maintained and our primary endpoint will remain overall survival. Two years is a commonly used minimum follow-up time for comparable oncological trials, which is expected to capture the most relevant data for the short-term analysis. Moreover, the short-term results of the trial and thus the potential implementation of active surveillance as an alternative treatment strategy can be performed a year earlier, avoiding the unnecessary delay of providing organ sparing treatment for patients with locally advanced oesophageal cancer. Importantly, long-term analyses will be performed after the last included patient finished the active surveillance protocol (minimum follow-up of 5 years), as was previously defined.

## Ethical and regulatory considerations

No amendments have been made to the ethical and regulatory considerations.

## Conclusion

In conclusion, substantial amendments were made prior to closure of enrolment of the SANO trial. These amendments do not affect the outcomes of the trial compared to the original protocol. The last patient with a clinically complete response is expected to be included in May 2021. Guaranteeing a minimum follow-up of 2 years, the first results are expected late 2023.

## Supplementary Information

**Additional file 1.**

## Data Availability

Individual patient data will not be made available.

## Abbreviations

18F-FDG: Fluorodeoxyglucose
cCR: Clinically complete response
CRE: Clinical response evaluation
CRE-1: First clinical response evaluation
CRE-2: Second clinical response evaluation
CROSS: Chemoradiotherapy for oesophageal cancer followed by surgery study
EUS: Endoscopic ultrasonography
nCRT: Neoadjuvant chemoradiotherapy
pCR: Pathologically complete response
PET/CT: Positron emission tomography with computed tomography
preSANO: Pre-surgery as needed for oesophageal cancer
preSINO: Pre-surgery if needed for oesophageal cancer
QoL: Quality of life
R1: Microscopically non-radical
RCT: Randomised controlled trial
SCC: Squamous cell carcinoma
SANO: Surgery As Needed for Oesophageal cancer
SINO: Surgery if needed for oesophageal cancer
TRG: Tumour regression grade

## References

[1] Noordman BJ, Wijnhoven BPL, Lagarde SM, Boonstra JJ, Coene P, Dekker JWT (2018). Neoadjuvant chemoradiotherapy plus surgery versus active surveillance for oesophageal cancer: a stepped-wedge cluster randomised trial. BMC Cancer.

[2] van Hagen P, Hulshof MC, van Lanschot JJ, Steyerberg EW, van Berge Henegouwen MI, Wijnhoven BP (2012). Preoperative chemoradiotherapy for esophageal or junctional cancer. N Engl J Med.

[3] Noordman BJ, Spaander MCW, Valkema R, Wijnhoven BPL, van Berge Henegouwen MI, Shapiro J, Biermann K, van der Gaast A, van Hillegersberg R, Hulshof MCCM, Krishnadath KK, Lagarde SM, Nieuwenhuijzen GAP, Oostenbrug LE, Siersema PD, Schoon EJ, Sosef MN, Steyerberg EW, van Lanschot JJB, Doukas M, Krak NC, Poley JW, van Rij CM, Bergman JJGHM, Gisbertz SS, van Laarhoven HWM, Meijer SL, Goense L, Haj Mohammad N, Hobbelink MGG, Offerhaus GJA, Vleggaar F, Curvers WL, Creemers GJ, Roef MJ, van der Sangen MJC, Buijsen J, Riedl RG, Schreurs WMJ, Warmerdam FARM, Janssen MJR, van der Post C, Radema SA, Rosman C, Rütten H (2018). Detection of residual disease after neoadjuvant chemoradiotherapy for oesophageal cancer (preSANO): a prospective multicentre, diagnostic cohort study. Lancet Oncol.

[4] Shapiro J, van Hagen P, Lingsma HF, Wijnhoven BP, Biermann K, ten Kate FJ, Steyerberg EW, van der Gaast A, van Lanschot J, CROSS Study Group (2014). Prolonged time to surgery after neoadjuvant chemoradiotherapy increases histopathological response without affecting survival in patients with esophageal or junctional cancer. Ann Surg.

[5] van der Werf LR, Dikken JL, van der Willik EM, van Berge Henegouwen MI, Nieuwenhuijzen GAP, Wijnhoven BPL, Bosscha K, van Grieken NCT, Hartgrink HH, van Hillegersberg R, Lemmens VEPP, Plukker JT, Rosman C, van Sandick JW, Siersema PD, Tetteroo G, Veldhuis PMJF, Voncken FEM (2018). Time interval between neoadjuvant chemoradiotherapy and surgery for oesophageal or junctional cancer: a nationwide study. Eur J Cancer.

[6] Shapiro J, van Lanschot JJB, Hulshof M, van Hagen P, van Berge Henegouwen MI, Wijnhoven BPL, van Laarhoven H, Nieuwenhuijzen GAP, Hospers GAP, Bonenkamp JJ, Cuesta MA, Blaisse RJB, Busch ORC, ten Kate F, Creemers GM, Punt CJA, Plukker JTM, Verheul HMW, Bilgen EJS, van Dekken H, van der Sangen M, Rozema T, Biermann K, Beukema JC, Piet AHM, van Rij C, Reinders JG, Tilanus HW, Steyerberg EW, van der Gaast A, CROSS study group (2015). Neoadjuvant chemoradiotherapy plus surgery versus surgery alone for oesophageal or junctional cancer (CROSS): long-term results of a randomised controlled trial. Lancet Oncol..

[7] van der Wilk BJ, Noordman BJ, Neijenhuis LKA, Nieboer D, Nieuwenhuijzen GAP, Sosef MN, et al. Active surveillance versus immediate surgery in clinically complete responders after neoadjuvant chemoradiotherapy for esophageal cancer: a multicenter propensity matched study. Ann Surg. 2019. 10.1097/SLA.0000000000003636. Epub ahead of print. 10.1097/SLA.0000000000003636 31592898

