# Supplementary material for: Updated protocol of the SANO trial: a stepped-wedge cluster randomised trial comparing surgery with active surveillance after neoadjuvant chemoradiotherapy for oesophageal cancer
Source: Trials. 2021 May 17;22:345. doi: 10.1186/s13063-021-05274-w (PMC8127221; doi:10.1186/s13063-021-05274-w)
Supplement: Supplementary file 1 — Additional file 1. [file 13063_2021_5274_MOESM1_ESM.pdf]

Neoadjuvant chemoradiotherapy plus surgery versus active surveillance for oesophageal cancer

(SANO trial: "Surgery As Needed for Oesophageal cancer")

**Protocol ID**	SANO
**Short title**	Surgery as Needed for Oesophageal cancer
**EudraCT number**	**NA**
**Version**	V1.7
**Date**	25-11-2020
**Coordinating investigators**	**Erasmus MC, Rotterdam**<br>drs. B.J. van der Wilk<br>Afdeling Heelkunde, Erasmus MC, Room Na-2119<br>P/O box, 3000 CA Rotterdam<br><br>drs. B.M. Eyck<br>Afdeling Heelkunde, Erasmus MC, Room Na-2119<br>P/O box 2040, 3000 CA, Rotterdam
**Principal investigators**	**Erasmus MC, Rotterdam**<br>prof. dr. J.J.B. van Lanschot<br>Afdeling Heelkunde, Erasmus MC, Room Rg-229<br>P/O box 2040, 3000 CA Rotterdam<br><br>dr. B.P.L. Wijnhoven<br>Afdeling Heelkunde, Erasmus MC<br>P/O box 2040, 3000 CA Rotterdam<br><br>dr. S.M. Lagarde<br>Afdeling Heelkunde, Erasmus MC<br>Postbus 2040, 3000 CA Rotterdam
**Sponsor:**	Erasmus MC
**Subsidising parties**	Ministry of Health, Welfare and Sport (ZonMW)<br>Koningin Wilhelmina Fonds (KWF) Kankerbestrijding
**Independent expert (s)**	Dr. B. Groot Koerkamp, hepato-pancreato-biliary surgeon<br>Afdeling Heelkunde, Erasmus MC<br>Postbus 2040, 3000 CA Rotterdam
**Laboratory sites**	**NA**
**Pharmacy**	**NA**
**Data Centre and Monitoring**	Clinical Trial Center – Erasmus MC Cancer Institute<br>Erasmus MC Rotterdam , room EE21-16<br>Dr. Molewaterplein 40, 3015 GD, Rotterdam, the Netherlands<br>P/O box 2040, 3000CA Rotterdam, The Netherlands

TABLE OF CONTENTS

SUMMARY ... 8
1. INTRODUCTION AND RATIONALE ... 10
2. OBJECTIVES ... 17
3. STUDY DESIGN ... 18
  3.1 Overall design ... 18
  3.2 Duration of the study ... 18
  3.3 Study Overview (Figure 4) ... 19
4. STUDY POPULATION ... 25
  4.1 Study population ... 25
  4.2 Inclusion criteria ... 25
  4.3 Exclusion criteria ... 25
  4.4 Sample size calculation ... 25
5. TREATMENT OF SUBJECTS ... 28
  5.1 Investigational product/treatment ... 29
  5.2 Use of co-intervention (if applicable) NA ... 32
  5.3 Escape medication (if applicable) NA ... 32
6. INVESTIGATIONAL PRODUCT ... 33
  6.1 Name and description of investigational product(s) NA ... 33
  6.2 Summary of findings from non-clinical studies NA ... 33
  6.3 Summary of findings from clinical studies NA ... 33
  6.4 Summary of known and potential risks and benefits NA ... 33
  6.5 Description and justification of route of administration and dosage NA ... 33
  6.6 Dosages, dosage modifications and method of administration NA ... 33
  6.7 Preparation and labelling of Investigational Medicinal Product NA ... 33
  6.8 Drug accountability NA ... 33
7. NON-INVESTIGATIONAL PRODUCT ... 34
  7.1 Name and description of non-investigational product(s) NA ... 34
  7.2 Summary of findings from non-clinical studies NA ... 34
  7.3 Summary of findings from clinical studies NA ... 34
  7.4 Summary of known and potential risks and benefits NA ... 34
  7.5 Description and justification of route of administration and dosage NA ... 34
  7.6 Dosages, dosage modifications and method of administration: ... 34
  7.7 Preparation and labelling of Non Investigational Medicinal Product NA ... 34
  7.8 Drug accountability NA ... 34
8. METHODS ... 35
  8.1 Study parameters/endpoints ... 35
  8.2 Randomisation, blinding and treatment allocation ... 35
  8.3 Study procedures ... 39
  8.4 Withdrawal of individual subjects ... 47
  8.5 Replacement of individual subjects after withdrawal ... 47
  8.6 Follow-up of subjects withdrawn from treatment ... 47
  8.7 Premature termination of the study ... 47

8.8 Expected outcome active surveillance arm 50
8.9 Proctoring 52
9. SAFETY REPORTING 53
9.1 Section 10 WMO event 53
9.2 AEs, SAEs and SUSARs 53
9.2.1 Adverse events (AEs) 53
9.2.2 Serious adverse events (SAEs) 53
9.2.3 Suspected unexpected serious adverse reactions (SUSARs) NA 54
9.3 Annual safety report NA 54
9.4 Follow-up of adverse events 54
9.5 Safety committee 55
9.6 Resonance group 55
10. STATISTICS 56
10.1 Primary study parameter(s) 56
10.2 Secondary study parameter(s) 56
10.3 Other study parameters NA 56
10.4 Interim analysis (if applicable) 56
11. ETHICAL CONSIDERATIONS 58
11.1 Regulation statement 58
11.2 Recruitment and consent 58
11.3 Objection by minors or incapacitated subjects NA 58
11.4 Benefits and risks assessment, group relatedness 58
11.5 Compensation for injury 59
12. ADMINISTRATIVE ASPECTS, MONITORING AND PUBLICATION 60
12.1 Handling and storage of data and documents 60
12.2 Monitoring and Quality Assurance 60
12.3 Amendments 60
12.4 Annual progress report 61
12.5 End of study report 61
12.6 Public disclosure and publication policy 61
13. STRUCTURED RISK ANALYSIS NA 62
13.1 Potential issues of concern NA 62
13.2 Synthesis NA 62
14. APPENDICES 63
15. REFERENCES 75

LIST OF ABBREVIATIONS AND RELEVANT DEFINITIONS

ABR	ABR form, General Assessment and Registration form, is the application form that is required for submission to the accredited Ethics Committee (In Dutch, ABR = Algemene Beoordeling en Registratie)
AE	Adverse Event
AR	Adverse Reaction
AUC	Area Under the Curve
BEV	Beam's Eye View
CA	Competent Authority
CCMO	Central Committee on Research Involving Human Subjects; in Dutch: Centrale Commissie Mensgebonden Onderzoek
cCR	Clinically Complete Response
cNCR	Clinically Non-Complete Response
CRE	Clinical Response Evaluation
CROSS	ChemoRadiotherapy for Oesophageal cancer followed by Surgery Study
CT	Computed Tomography
CTV	Clinical Target Volume
CV	Curriculum Vitae
DVH	Dose Volume Histogram
DFS	Disease Free Survival
DUCA	Dutch Upper-GI Cancer Audit
EANM	European Association for Nuclear Medicine
EARL	EANM Research Ltd
ECG	ElectroCardioGram
ECCG	Esophagectomy Complications Consensus Group
eGFR	Estimated Glomerular Filtration Rate
EORTC	European Organisation for Research and Treatment of Cancer
EQ-5D	EuroQol 5 Dimensions
EU	European Union
EUS	Endoscopic UltraSonography
18F-FDG	^{18}F-FluDeoxyGlucose
FNA	Fine Needle Aspiration
GFR	Glomerular Filtration Rate
GCP	Good Clinical Practice
GTV	Gross Tumour Volume
Gy	Gray
HR	Hazard Ratio
IB	Investigator's Brochure
IC	Informed Consent
ICRU	International Commission on Radiation Units
IMP	Investigational Medicinal Product
IMPD	Investigational Medicinal Product Dossier
IMRT	Intensity Modulated RadioTherapy
METC	Medical research ethics committee (MREC); in Dutch: medisch ethische toetsing commissie (METC)
MTB	Multidisciplinary Tumour Board
MTT	Maximum tumour thickness

mSV	MilliSievert
MV	MegaVolt
NA	Not Applicable
nCRT	neo-adjuvant ChemoRadioTherapy
NTR	Netherlands Trial Registry
OGD	OesophagoGastroDuodenoscopy
OS	Overall Survival
pCR	pathologically Complete Response
PET-CT	Positron Emission Tomography – Computed Tomography
preSANO	Pre-Surgery As Needed for Oesophageal Cancer
PTV	Planning Target Volume
QALY	Quality Adjusted Life Year
QLQ	Quality of Life Questionnaire
R_0	Microscopically Radical resection
R_1	Microscopically irradical resection
R_2	Macroscopically irradical resection
(S)AE	(Serious) Adverse Event
SANO	Surgery As Needed for Oesophageal cancer
SPC	Summary of Product Characteristics (in Dutch: officiële productinfomatie IB1-tekst)
Sponsor	The sponsor is the party that commissions the organisation or performance of the research, for example a pharmaceutical company, academic hospital, scientific organisation or investigator. A party that provides funding for a study but does not commission it is not regarded as the sponsor, but referred to as a subsidising party.
SUSAR	Suspected Unexpected Serious Adverse Reaction
SUV	Standardised Uptake Value
TNM	Tumour Node Metastasis classification system
TOF	Time Of Flight
TRG	Tumour Regression Grade
UICC	Union for International Cancer Control
US	UltraSound
Wbp	Personal Data Protection Act (in Dutch: Wet Bescherming Persoonsgevens)
WMO	Medical Research Involving Human Subjects Act (in Dutch: Wet Medisch-wetenschappelijk Onderzoek met mensen)
ypTNM	Pathological Tumour Node Metastasis classification system post-neoadjuvant treatment

SUMMARY

Rationale: We propose an active surveillance approach after completion of neoadjuvant chemoradiotherapy (nCRT) for carcinoma of the oesophagus. In this *SANO* (*i.e.* Surgery As Needed for Oesophageal cancer) approach, surgical resection is offered only to patients in whom a locoregional regrowth is highly suspected or proven, without distant dissemination. Such an organ-preserving strategy can have great advantages, but is only justified if long-term survival is non-inferior to that of the current standard trimodality approach comprising neoadjuvant chemoradiotherapy followed by standard surgery.

Objective: The aim of this study is to assess the (cost-)effectiveness (including non-financial costs and survival) of active surveillance for patients with squamous cell- or adenocarcinoma of the oesophagus or oesophago-gastric junction.

Study design: phase III multi-centre, stepped-wedge cluster randomised controlled trial.

Study population: Operable patients ≥ 18 years of age with potentially curable locoregionally advanced squamous cell- or adenocarcinoma of the oesophagus or oesophago-gastric junction.

Intervention (if applicable): Approximately 4-6 weeks after completion of nCRT all patients will undergo a first clinical response evaluation (CRE-I) consisting of endoscopy with (random) bite-on-bite biopsies of the primary tumour site and of any other suspected laesions in the oesophagus. Patients who are clinically complete responders (i.e. patients without local or disseminated disease proven by histology) will undergo a second clinical response evaluation (CRE-II), 6-8 weeks after CRE-I (i.e. 10-14 weeks after completion of nCRT). CRE-II will include a whole body 18F-FDG PET-CT, followed by endoscopy with (random) bite-on-bite biopsies of the primary tumour site and any other suspected laesions in the oesophagus and linear EUS plus FNA of suspected lymph nodes. Patients who have a clinically complete response after CRE-II will be assigned to either surgical resection or active surveillance. During the first phase of the study, these patients will undergo surgical resection, which is standard practice. After this phase, centres will change their policy to active surveillance, with the duration of the first phase determined randomly over the 12 centres (*i.e.* stepped wedge cluster design). Patients enrolled in the active surveillance arm will undergo diagnostic evaluations similar to CRE-II every 3 months in the first year after completion of neoadjuvant treatment, every 4 months in the second year, every 6 months in the third year and yearly in the 4th and 5th year of follow up, or when symptoms or results of any diagnostic test require shorter assessment intervals. In the active surveillance arm, surgical resection will be offered only to those patients, in whom a locoregional regrowth is highly suspected or proven, without any signs of distant dissemination.

Main study parameters/endpoints: the main study parameter is overall survival; secondary endpoints include the percentage of patients who do not undergo surgery, quality of life, clinical irresectability (cT4b) rate, radical resection rate, postoperative complications, progression free survival, distant dissemination rate, and cost-effectiveness.

Nature and extent of the burden and risks associated with participation, benefit and group relatedness: the main burden for participating patients are the additional diagnostic tests as part of the CREs and surveillance evaluations after completion of nCRT. The number and frequency of diagnostic rounds depend on whether residual disease is detected. The CREs and surveillance evaluations consist of PET-CT, endoscopy and endoscopic ultrasonography (EUS) with or without fine needle aspiration (FNA). All three tests carry a minimal risk of

complications. The main risk for patients in the active surveillance arm is that tumour regrowth is detected beyond the resectability limit, that distant metastases develop from local regrowths and that delayed surgery is potentially associated with an increase in postoperative morbidity. Therefore, an intensive surveillance regimen has been incorporated and strict stopping rules for ensuring patients oncological safety have been formulated for this trial. Participating patients may benefit by the avoidance of an oesophagectomy, which is associated with severe morbidity, substantial postoperative mortality and impact on patients' quality of life. Moreover, it is expected that ultimately health care costs will substantially decrease by active surveillance.

1. INTRODUCTION AND RATIONALE

1.1 Oesophageal cancer

Cancer of the oesophagus and oesophago-gastric junction is a highly lethal malignancy, as reflected by an average overall 5-year survival of 17% (1). In the Netherlands, the incidence of oesophageal cancer resembles the growing trend in Western countries, with an estimated incidence of 15/100,000 for men and 6/100,000 for women , and more than 2600 new cases diagnosed annually. (2)

1.2 Surgical treatment with curative intent

Presently, surgical resection is considered the cornerstone of curative treatment in the Netherlands for stages cT1b-4aN0-3M0 oesophageal or junctional cancer. In the international literature, reported 5-year survival rates for patients treated with primary surgical resection range from 6 to 50%, but rarely exceed 35%. (3-7) However, oesophageal resections are associated with postoperative mortality rates of 1-5% in high-volume centres, severe postoperative morbidity and a substantial impact on the quality of life . (8-13) In order to improve the radicality of surgical resection and long-term survival, many trials on the added value of (neo-) adjuvant chemo- and/or radiotherapy have been undertaken. (14-17) One of the largest and most successful of these trials is the Dutch CROSS trial (ChemoRadiotherapy for Oesophageal cancer followed by Surgery Study), which compared neoadjuvant chemoradiotherapy plus surgery to surgery alone. (18)

1.3 CROSS trial

The CROSS trial is a multicentre, randomised, controlled Dutch clinical trial (18). The study included and analysed 366 patients during a 5-year period from 5 academic and 2 non-academic teaching hospitals in the Netherlands. The study compared neoadjuvant chemoradiotherapy followed by surgery with surgery alone in patients with potentially curable advanced squamous cell carcinoma or adenocarcinoma of the oesophagus or oesophago-gastric junction (cT2-3 N0-1 M0 and cT1 N1 M0, according to the UICC TNM classification, 6th edition), with a planned inclusion of 175 patients per arm. The neoadjuvant regimen consisted of Carboplatin (AUC = 2) and Paclitaxel (50 mg/m^2) given by intravenous infusion on days 1, 8, 15, 22 and 29, combined with concurrent radiation therapy using a multiple field technique. A total dose of 41.4 Gy was given in 23 fractions of 1.8 Gy, 5 fractions per week, starting on the first day of the first cycle of chemotherapy. Treatment related toxicity was low compared to other (neo)adjuvant and definitive chemo(radio)therapy regimens, with 95% of all patients who received any neoadjuvant chemoradiotherapy able to complete the entire regimen. Median overall survival of patients who received neoadjuvant chemoradiotherapy plus surgery was 49 months, compared to 24 months for those who received surgery alone and the 5-year overall survival was superior in the neoadjuvant chemoradiotherapy arm (HR = 0.68; 95% confidence interval 0.53-0.88; *P* = .003). (19)

In conclusion, results from the CROSS trial show that the addition of neoadjuvant chemoradiotherapy (Carboplatin, Paclitaxel and 41.4 Gy concurrent radiotherapy) to surgery significantly increases long-term survival as compared to surgery alone. Therefore, neoadjuvant chemoradiation plus surgery is now

considered the therapy of first choice in the Netherlands and around 80% of patients with oesophageal cancer in a potentially curative setting undergo this trimodality treatment. Nevertheless, still approximately 50% of these patients develop haematogenous metastases, mostly (>90% of patients) within 2 years after surgery. (18-20)

1.4 Pathologically complete response

In subsequent analyses of secondary endpoints of the CROSS trial it was found that nearly a third of the patients had a pathologically complete response in the resection specimen. This means that no viable tumour cells were found at the site of the primary tumour or in the resected regional lymph nodes, as determined by conventional histological examination. A pathologically complete response after neoadjuvant chemoradiotherapy was seen in 49% of patients with a squamous cell carcinoma and 23% of patients with an adenocarcinoma. This observation raises the question if a surgical resection was of benefit to these patients and if patients already were cured locoregionally by this potent neoadjuvant treatment alone. Theoretically, an organ sparing approach might be feasible since intuitively an oesophagectomy in patients with no residual viable tumour cells likely does not change the outcome, but only puts the patient at risk for perioperative mortality and morbidity and reduces quality of life in the short and long term. Hence this imposes an ethical imperative to reconsider the necessity of standard oesophagectomy in patients after neoadjuvant chemoradiotherapy. An individualised approach to surgery after neoadjuvant chemoradiotherapy should be studied and defined; a new treatment algorithm in which not every patient with potentially curable oesophageal cancer needs a resection after completion of neoadjuvant chemoradiotherapy to achieve long-term survival. Hence, a *surgery as needed* (SANO) approach after completion of neoadjuvant chemoradiotherapy for carcinoma of the oesophagus is proposed.

1.5 Active surveillance

In this SANO approach, patients will undergo active surveillance after completion of neoadjuvant chemoradiotherapy. Surgical resection will be offered <u>only</u> to patients in whom a locoregional regrowth is highly suspected or proven, without signs of distant dissemination.

In other types of cancer such as rectal cancer and head and neck cancer, similar approaches have been evaluated with excellent results. (21-24) In a recent study in patients who underwent neoadjuvant chemoradiotherapy for rectal cancer, 65% of all patients with a clinically complete response (cCR) did not need surgery. Of the remaining 35%, 90% were successfully operated by delayed surgical resection and oncological outcome was comparable between both treatment groups.(22) In advanced head and neck cancer active surveillance after chemoradiotherapy is widely accepted nowadays. In a recent randomised controlled trial (RCT) that compared standard neck dissection with PET-CT guided surveillance, survival was similar in both treatment groups, but surveillance resulted in considerably fewer operations and was more cost-effective. (24)

Clinical response evaluations: identifying and excluding pathologically non-responders or minor responders

Before including patients into an active surveillance protocol, patients with locoregional residual disease after completion of neoadjuvant chemoradiotherapy have to be identified and excluded. In these patients – under the condition that no signs of disseminated disease are present - there is no benefit of delaying surgery after completion of neoadjuvant therapy, since surgery remains the only potentially curative treatment for these patients.

Therefore, after registration in this SANO trial and subsequent neoadjuvant chemoradiotherapy, all patients will be re-evaluated once by endoscopy with bite-on-bite biopsies (CRE-I) and if negative once by PET-CT, endoscopy with bite-on-bite biopsies and endoscopic ultrasound with fine-needle aspiration (FNA) (CRE-II, Figure 1).

The aim of these assessments is to identify and exclude patients with residual and/or disseminated disease. Patients are categorised as clinically complete responders or clinically non-complete responders. If distant metastases are detected during a clinical response evaluation, that patient is not eligible for participation and will be referred for palliative care. Only clinically complete responders (*i.e.* patients in whom no locoregional or disseminated disease can be proven) will be offered inclusion into the randomised part of this trial (Figure 1).

Figure 1 - **SANO trial design**

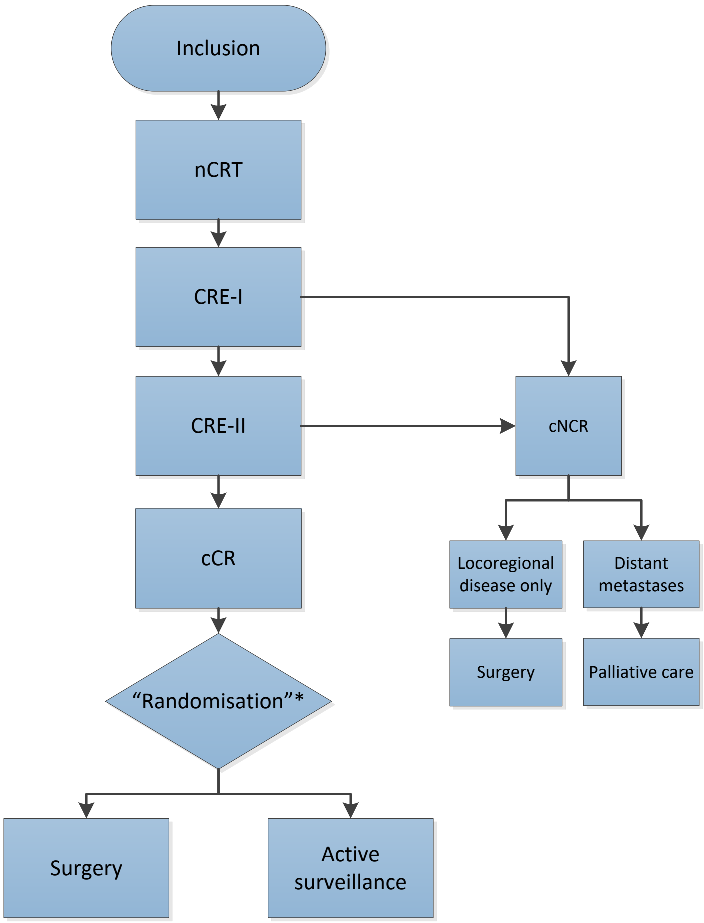

nCRT: neoadjuvant chemoradiotherapy; CRE: clinical response evaluation; cNCR: clinically non-complete response; cCR: clinically complete response. *At this point the patient will be allocated to one of the two treatment arms, dependent on the institution in which the actual treatment takes place. Randomisation has already been performed at institutional level (see §3.1 and §8.2) and will be known already at the moment of inclusion.

Who benefits from the active surveillance approach?

An organ-preserving SANO-strategy in oesophageal cancer will not only have advantages for individuals who are cured by neoadjuvant therapy alone, but also for patients with subclinical disseminated disease (*i.e.* micrometastases) at the time of completion of neoadjuvant therapy. After tumour staging and neoadjuvant treatment, micrometastases may be present but non-detectable. With time these disseminated tumour cells can become clinically manifest. Distant metastases, which are the main determinants of long-term survival after neoadjuvant chemoradiation plus surgery (esp. in patients with a pathologically complete response), are grossly independent of locoregional therapy. (25, 26) Although the biology of distant dissemination is not fully understood, current assumptions hold that the process of spreading and seeding of tumour cells from the primary laesion is an early event and thus has already occurred in many patients at the time of first clinical presentation and subsequent locoregional treatment (*i.e.* neoadjuvant chemoradiation with and without subsequent surgery). (27) This is reflected by the large number of patients who develop haematogenous metastases within 2 years after surgery. (18-20) No matter how timely and aggressive locoregional treatment will be, it will hardly affect the survival-determining events of distant dissemination. At present, patients with

occult distant metastases undergo a non-beneficial pseudo-curative oesophageal resection because the metastases are below the detection limit at the first clinical evaluation after neoadjuvant chemoradiotherapy. A SANO approach is only justified if long-term oncological outcome is comparable to neoadjuvant chemoradiotherapy followed by surgery. Therefore, tumour regrowth after neoadjuvant chemoradiotherapy should be detected at a curable stage, *i.e.* in the time window between the *clinical detection limit** and the *resectability limit* (between interrupted vertical green lines, Figure 2) and before the development of distant dissemination from disease regrowth. Currently, it is not known what the length of time of this window of opportunity and the variation between patients are. Therefore, an intensive surveillance regimen is proposed, aiming to detect as many regrowths, as early as possible before they become irresectable. Since the majority of locoregional regrowths are expected to occur within 12 months, and nearly all within 24 months, this surveillance regimen should be most intensive in the first 2 years. (28)

* The clinical detection limit is the minimal amount of disease that can be detected by the combination of symptoms, endoscopy with (bite-on-bite) biopsies and imaging modalities.

Figure 2 - **Tumour response after neoadjuvant chemoradiotherapy**

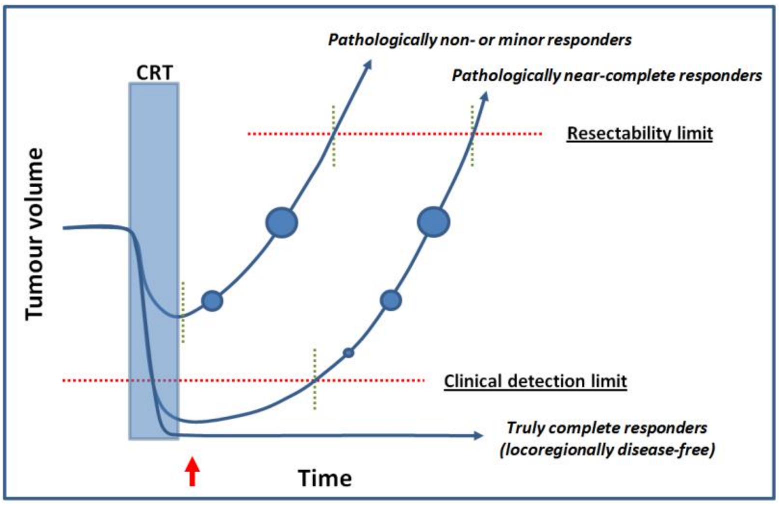

CRT: chemoradiotherapy; Red arrow: time of clinical response evaluation (CRE); Vertical interrupted green lines: boundaries of theoretical time windows. First vertical interrupted green line on each curve refers to the first moment after CRT that a tumour becomes clinically detectable. Second vertical interrupted green line on each curve refers to the moment that a tumour becomes irresectable (T4b). Circles depict progression of locoregional tumour volume.

1.6 Feasibility of the SANO-approach

In recent years, multiple studies focussed on the accuracy of detecting residual disease after neoadjuvant chemoradiotherapy for oesophageal cancer, however surgical resection as standard treatment in a potentially curative setting was (almost) always performed.

Although the accuracy of endoscopy with standard biopsies is limited (29), a recent study from Dublin suggested that endoscopy with deep (bite-on-bite) biopsies is significantly more accurate in detecting residual disease after neoadjuvant chemoradiotherapy. Negative bite-on-bite biopsies were 85% predictive for a pathologically complete response in the resection specimen (*i.e.* 15% false negative for any residual cancer). (30) Bite-on-bite biopsies increase the chance of detecting residual submucosal tumour deposits compared to conventional biopsies. After neoadjuvant chemoradiotherapy, residual disease is frequently located in the

submucosa (and mucosa), and rarely as an isolated remnant only in the proper muscle layer, surrounding stroma or regional lymph nodes. (31) The use of fine-needle aspiration to detect lymph node metastases in patients with a complete response in the primary tumour may further increase the diagnostic accuracy. 18F-FDG PET-CT is able to distinguish non- or minor responders (>10% residual vital tumor) from major- and complete responders (≤10% vital residual tumour). (32, 33) After neoadjuvant chemoradiotherapy 18F-FDG PET-CT cannot rule out microscopic residual disease in near-complete responders (1-10% residual vital tumour), but in the SANO approach we expect these tumours to become detectable by the diagnostic modalities used during active surveillance as soon as they progress during follow up, before they have become irresectable. This hypothesis is supported by the available literature on active surveillance after nCRT for oesophageal cancer, showing that a delayed resection can be performed successfully in nearly all patients with residual locoregional disease that has been missed initially during response evaluation using endoscopy with (conventional) biopsies and PET-CT (see below). (34-36)

The maximum tumour thickness (MTT) as determined by endoscopic ultrasound (EUS) is predictive for histopathological response on neoadjuvant chemoradiotherapy. A Swiss study found significant correlations of pathologically complete response with an absolute MTT ≤6 mm (P = .008) and a relative change in thickness (ratio response measurement/baseline examination) ≤50% (P = .003). (37)

The recently published preSANO trial aimed to investigate the optimal set of diagnostics to detect residual disease in patients that underwent neoadjuvant chemoradiotherapy for oesophageal cancer (38). Results of this study revealed that a combination of endoscopic bite-on-bite biopsies, EUS with fine-needle aspiration (FNA) of suspected lymph nodes and 18F-FDG PET-CT had a sensitivity of 90% to detect TRG3-4 residual tumours (>10% residual tumour cells). Furthermore, these diagnostic modalities are safe to use after neoadjuvant chemoradiotherapy (one small mucosal tear without any clinical consequences and one cardiac arrhythmia after endoscopy, unrelated to the endoscopy and without any clinical consequences have been reported). Nine percent of patients reveal interval metastases during the first two CREs. Approximately 40% of the patients that underwent complete CREs followed by surgery in preSANO-2 were considered to have a clinically complete response.

(39)

The safety of delaying surgery in participants of the SANO approach is supported by a recent study suggesting that prolonged time to surgery after neoadjuvant chemoradiotherapy up to at least 12 weeks has no effect on disease-free and overall survival (HR=1.00 and HR=1.06 per additional week, P=.976 and P=.139, respectively). Moreover, prolonged time to surgery increases the probability of a pathologically complete response in the resection specimen (odds ratio = 1.35 per additional week of time to surgery, P=.0004). (40) Comparable results have been published by other groups. (41-43) Consequently, several national and international centres have started to postpone surgical resection after completion of neoadjuvant therapy to allow individual patients to maximise recovery after neoadjuvant therapy, before proceeding to surgery.

Finally, a few small retrospective studies support an active surveillance approach in selected patients with cCR after neoadjuvant chemoradiotherapy for oesophageal cancer. Some 61 patients from MD Anderson Cancer Center who declined surgery after neoadjuvant chemoradiotherapy and cCR based on PET-CT and

endoscopy with conventional biopsies showed a 5-year overall survival rate of 58%. Moreover, 12 of 13 patients with local regrowth during surveillance underwent a successful (salvage) oesophagectomy. (34) In a subsequent comparative analysis, 36 active surveillance patients were matched using the propensity-score method to 36 patients who underwent neoadjuvant chemoradiotherapy followed by standard surgery. Estimated median overall survival was (non-significantly) better in the active surveillance group than in the standard surgery group (58 vs. 51 months, respectively, p=0.28). All 11 patients with locoregional regrowth in the active surveillance group underwent delayed surgery with excellent outcome (median overall survival 58 months). Furthermore, distant dissemination rate was similar in both groups (31% in the active surveillance group and 28% in the standard surgery group). (35) A similar Italian study compared standard surgery (n=39) with active surveillance (n=38) in patients with cCR after neoadjuvant chemoradiotherapy for oesophageal squamous cell carcinoma. Clinical response was assessed using endoscopy with conventional biopsies only and patients in the surveillance group were not operated on because they were considered unfit for surgery or declined surgery. Nevertheless, 5-year overall survival rates were comparable in both groups (50.0 % in the surgery group vs. 57.0 % in the active surveillance group, p=0.99). (36) Similar results were described in two small Irish studies that analysed 92 and 25 patients, respectively, who underwent neoadjuvant chemoradiotherapy +/- surgery in case of cCR on endoscopy post-neoadjuvant chemoradiotherapy. (30, 44) Based on these promising results, an active surveillance strategy after neoadjuvant chemoradiotherapy for oesophageal cancer is currently offered to selected patients by several centres, such as MD Anderson Cancer Center. Taken together, these preliminary results suggest that a SANO approach is feasible, safe and efficacious, and can be tested safely in a large clinical trial.

2. OBJECTIVES

Primary objective

The main objective is to compare overall survival of an active surveillance approach to that of standard surgery for patients with either squamous cell- or adenocarcinoma of the oesophagus or oesophago-gastric junction

Secondary objectives

Secondary study objectives are:

- To assess the percentage of patients in the active surveillance arm who do not undergo surgery (*i.e.* patients who are cured by neoadjuvant chemoradiotherapy or who have occult distant metastases during initial staging, which become manifest during active surveillance);
- To compare quality of life between the arms;
- To assess cCR rate during CREs and surveillance evaluations;
- To assess the rate of locoregional regrowth, and tumour regrowth beyond the resectability limit in both arms;
- To compare the R_0-resection rates between the arms;
- To assess the safety of delayed surgical resection in the active surveillance arm;
- To assess the rate of distant dissemination in both arms;
- To analyse cost-effectiveness of an active surveillance strategy.

3. STUDY DESIGN

3.1 Overall design

Phase III multi-centre, stepped-wedge, cluster randomised controlled trial.

Trials randomising between surgical and non-surgical treatment modalities at a patient level frequently fail due to low accrual. (45-48) This can be explained by the fact that most patients have strong preferences regarding the choice to undergo surgery or not, and they are not willing to be randomised for such drastic strategies. Therefore, a stepped-wedge cluster design is preferred in the present trial. (49) This design involves sequential crossover of clusters of participating institutions from control (standard surgery) to intervention (active surveillance). Randomisation is performed at institutional level, instead of at individual level (Figure 3). (50)

Figure 3 - Example of stepped-wedge design for 4 institutions with 5 time periods

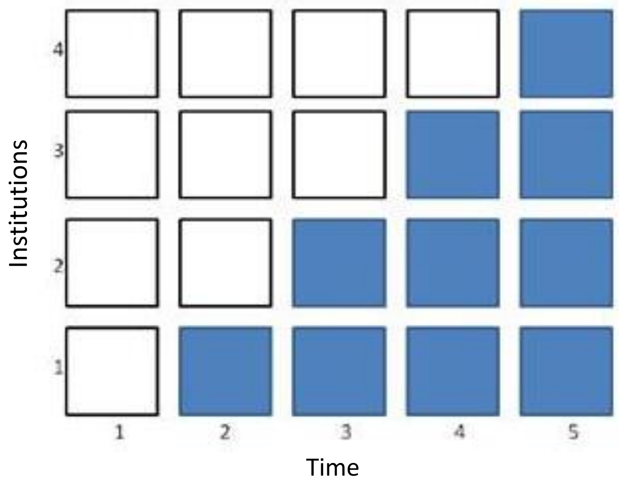

White boxes: control group (standard surgery), blue boxes: intervention group (active surveillance)

3.2 Duration of the study

The study is planned to include 140 patients in each arm (see §4.4). The inclusion period is estimated to last in total approximately 36 months. Initially, this estimation was based on a total of 600 resections for oesophageal cancer in all participating centres per year (as performed in 2015); 80% of patients (480 per year) underwent neoadjuvant chemoradiotherapy according to CROSS; we expected an inclusion rate of one out of three patients (160 per year). After one year of inclusion, preliminary results suggest that an inclusion rate of 22 per month (264 per year) is realistic. Because preSANO-2 reported a higher sensitivity than preSANO-1, the cCRs included in preSANO-1 will not be included in the SANO-trial. Furthermore, the CREs improved as such, that 34% instead of 50% of patients have a cCR. Consequently, the estimated inclusion rate increased (Figure 6). However, the duration of the study will not need to be extended as suggested by the inclusion rate so far. In order to determine the primary endpoint of overall survival with a minimum follow-up of 2 years, the study period will end two years after the last patient has been included. Patients will remain in follow-up for at least three more years after the end of the formal study.

Completion of the study

Since the clinically complete response rate and the rate of cross-over is variable (see §4.4), it is not possible to determine an exact number of patients that need to be included at baseline to end up with exactly the correct number of clinically complete responders. Therefore, to ensure that we do not end up with a sample size that is too small to maintain our predefined power of 80% with a significance of 0.05, we will continue including patients until we reach the predetermined 112 clinically complete responders in each arm. As a result, a limited number of patients will be included in the SANO-trial but will not be randomised yet (i.e. between inclusion and CRE-II, see Figure 1), while the inclusion of that particular arm will be completed. Patients already included will be offered surgical resection (= standard treatment) with minimal additional delay, or to proceed in active surveillance in case of clinically complete response. Since these patients already chose for active surveillance and gave informed consent for the active surveillance arm of the trial, it is expected that very few / none of these patients will prefer to stop active surveillance. Therefore, these patients will continue within the trial and will be included in the analysis of the SANO trial to increase the power of the trial.

3.3 Study Overview (Figure 4)

- We aim to include a total of 738 patients with squamous cell- or adenocarcinoma of the oesophagus or oesophago-gastric junction.
- Patients who underwent or are planned to undergo neoadjuvant chemoradiotherapy according to CROSS and are planned to undergo potentially curative surgical resection for histologically proven oesophageal or junctional squamous cell carcinoma or adenocarcinoma are eligible. Whenever pathology is inconclusive but a multidisciplinary expert group concludes oesophageal carcinoma because of radiologically or endosonographically highly suspected lesions, patients are eligible for the study.
- Patients will undergo conventional pre-treatment work-up (incl. an 18F-FDG PET-CT to assess the avidity of the primary tumour); only patients with FDG-avid tumour will be included in this trial. All included patients will receive neoadjuvant chemoradiotherapy according to the CROSS-protocol (18).
- Approximately 4-6 weeks after completion of neoadjuvant chemoradiotherapy all included patients will undergo a first clinical response evaluation (CRE-I) including oesophagogastroduodenoscopy (OGD) with at least 4 bite-on-bite biopsies of the primary tumour site and of any other suspected laesions in the oesophagus. Patients who are found to be clinically complete responders (*i.e.* those patients in whom no locoregional or disseminated disease can be proven by histology) will undergo a second clinical response evaluation (CRE-II) 6-8 weeks after CRE-I (i.e. 10-14 weeks after completion of nCRT). CRE-II will include an 18F-FDG PET-CT, followed by OGD with (random) bite-on-bite biopsies of the primary tumour site and any other suspected laesions in the oesophagus, and linear EUS plus FNA of all suspected lymph nodes, even if these lymph nodes are located directly behind the primary tumour site.* The PET-CT during CRE-II must be available to guide the endoscopist in taking targeted biopsies during OGD and EUS. **

- Patients with (cyto)histological evidence of locoregional residual disease during CRE-I, will be offered a subsequent 18F-FDG PET-CT to exclude disseminated disease and will be offered immediate surgery (*i.e.* 6-8 weeks after completion of neoadjuvant chemoradiotherapy).
- Patients with (cyto)histological evidence of locoregional residual disease or highly suspected locoregional residual disease on 18F-FDG PET-CT, and without distant metastases during CRE-II will undergo (postponed) surgery immediately after CRE-II (*i.e.* 12-14 weeks after completion of neoadjuvant chemoradiotherapy). Patients with distant metastases will be referred for palliative care.
- Patients without (cyto)histological evidence of residual disease and without highly suspected locoregional disease on 18F-FDG PET-CT during CRE-II, in the absence of distant metastases, will be randomised at institutional level to active surveillance or standard surgery (stepped-wedge design, see Study design).
- Patients who withdraw due to cross-over between treatment arms (*i.e.* patients allocated to the standard surgery arm who refuse to undergo surgery and patients allocated to the active surveillance arm who request immediate surgery in the absence of suspected locoregional disease) will be registered in the database to allow for per protocol comparative analysis. We recommend follow-up as described below for patients who withdraw due to cross-over from standard surgery to active surveillance. Follow-up for patients who withdraw due to cross-over from active surveillance to standard surgery is recommended to be performed according to the Dutch national guidelines for oesophageal cancer.
- Patients in the active surveillance arm will undergo active surveillance by PET-CT, OGD with at least 8 biopsies, (4 bite-on-bite biopsies) and EUS (+/- FNA) every 3 months in the first year after completion of neoadjuvant therapy, every 4 months in the second year, every 6 months in the third year and yearly in the 4th and 5th year of follow up, or when symptoms or results of any diagnostic test require shorter assessment intervals.* Surgical resection will be offered <u>only</u> to those patients, in whom a locoregional regrowth is highly suspected or proven, without any signs of distant dissemination (Figure 4).
- Patients in the standard surgery arm will be offered surgery immediately after CRE-II (*i.e.* 12-14 weeks after completion of neoadjuvant chemoradiotherapy). Follow-up of patients in the standard surgery arm consists of outpatient clinic visits at 6, 9, 12, 16, 20, 24, 30, 36, 48, 60 months after completion of neoadjuvant chemoradiotherapy. In order to accurately compare distant dissemination rates between both treatment arms, 18F-FDG PET-CT scan will be performed in all patients in the standard surgery arm after 16 and 30 months after completion of neoadjuvant chemoradiotherapy. , after which most (>80% and >90%, resp.) distant metastases will likely have been detected. (20)
- Patients with (cyto)histological evidence of disseminated disease during CRE-I, CRE-II or active surveillance will be excluded from further curative therapy and will be referred for palliative care (Figure 1 and 4).

* Cytology using FNA will also be obtained from lymph nodes that are located directly behind the primary tumour site, because the purpose of CRE-II is to detect any residual tumour, regardless of whether it is located at the primary tumour site or in the regional lymph nodes. Consequently, contamination is not an issue.

NB During CRE-I and CRE-II, positive (cyto)histology is preferably available when offering a patient surgical resection. However, during active surveillance we do allow a centralised multidisciplinary tumour board (MTB, Erasmus MC) to recommend surgical resection in selected patients that have a high clinical / diagnostic suspicion of tumour regrowth, despite repeatedly negative (cyto)histology. This centralised MTB will monitor and decide on all such suspected patients from all participating centres. Also, participating centres are obliged to present patients with high clinical /diagnostic suspicion of tumour regrowth without negative (cyto)histology to the MTB of the Erasmus MC if surgical resection is being considered. The reason for offering surgical resection in patients with a (strong) clinical suspicion of regrowth, but without positive (cyto)histology is to minimise the risk that a difficulty in confirming a regrowth by histology causes a delay that will permit a tumour regrowth to expand into an irresectable stage. If for instance the intensity of a hotspot on PET-CT increases over time during surveillance but positive (cyto)histology cannot be obtained, the MTB can decide to recommend surgery. Especially for tumours located proximally from the carina, a small increase of the intensity of the hotspot on PET-CT compared to CRE-II should result in immediate surgery, despite the absence of positive (cyto)histology, to avoid ingrowth of the primary tumor in the trachea.

Figure 4 - Study algorithm for active surveillance-arm

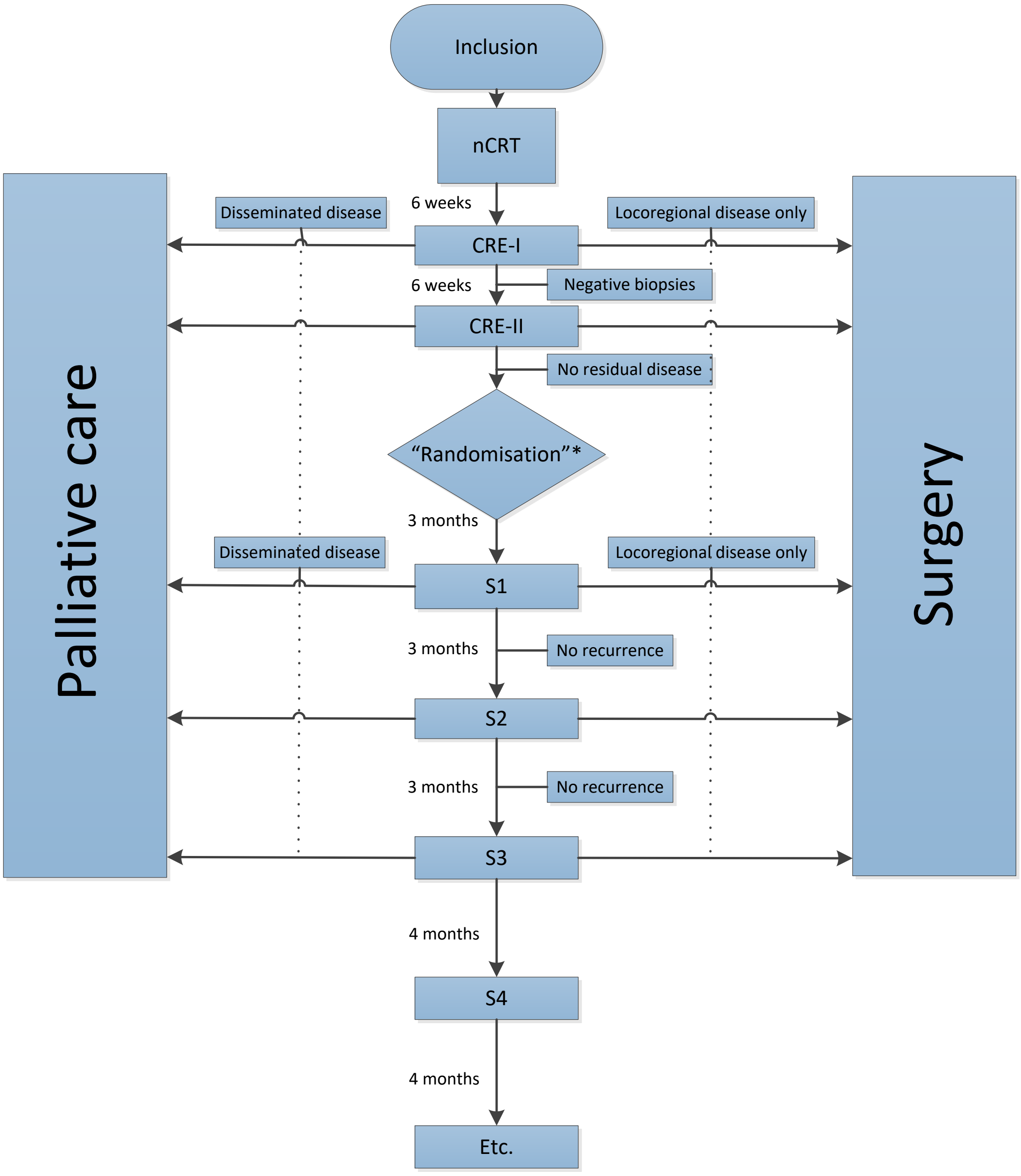

nCRT: neoadjuvant chemoradiotherapy; CRE: clinical response evaluation; S1: first surveillance evaluation; S2: second surveillance evaluation etc. Randomisation*: randomisation will be performed at institutional level (see §3.1 and §8.2) and will be known already at the moment of inclusion; immediate surgery arm of randomisation not shown.

3.4 Schedule of assessments

Parameter	Pretreatment	Neoadjuvant chemoradiotherapy (CROSS)	CRE-I	CRE-II (3 months after end of neoadjuvant chemoradio-therapy)	Active surveillance evaluations (6, 9, 12, 16, 20, 24, 30, 36, 48, 60 months after completion of neoadjuvant chemoradiotherapy[15])
**Eligibility check**	X				
**Written Informed consent**	X[10]				
**Inclusion**	X				
**"Randomisation" (treatment allocation)**				X[14]	
**Medical History**	X	X	X	X	X
**Physical Exam**	X	X	X	X	X
**ECOG Performance status** (Appendix B)	X	X	X	X	X
**Haematology**[1]	X	X			
**eGFR**	X	X			
**Biochemistry**[2]	X	X			
**Endoscopy + (random) bite-on-bite biopsies**	X		X	X	X
**Linear/Radial EUS (+FNA)**[4]	X			X	X
**CT of neck, thorax, abdomen and pelvis**	X				
**PET-CT (whole-body)**[18]	X		X[8]	X[9]	X[9]
**Pulmonary function tests**[5]	X				
**Bronchoscopy**[6]	X				
**ECG**	X				
**Toxicity**[7]	Baseline	X			
**Quality of Life (EQ-5D, QLQ-C30, QLC-OG25 and Cancer Worry Scale)**	X			X	X[16]
**Surgery**			X[11]	X[12]	X[13]
**Postoperative complications**				X[12]	X[13]

Parameter	Pretreatment	Neoadjuvant chemoradiotherapy (CROSS)	CRE-I	CRE-II (3 months after end of neoadjuvant chemoradio-therapy)	Active surveillance evaluations (6, 9, 12, 16, 20, 24, 30, 36, 48, 60 months after completion of neoadjuvant chemoradiotherapy[15])
**Pathology of resection specimen**				X[12]	X[13]
**Additional Blood[17]**	X		X	X	X

[1] Haematology: CBC, differential
[2] Biochemistry: serum protein, albumin, magnesium, electrolytes, serum creatinin, bilirubin, alkaline phosphatase, AST
[4] Linear EUS: with fine-needle aspiration (FNA) of any suspected lymph nodes
[5] Pulmonary function test: only on indication
[6] Bronchoscopy: when tumour is located above the carina and when there is suspicion for invasion of the tracheo-bronchial tree
[7] Toxicity: to be evaluated after each cycle
[8] PET-CT: during CRE-I, after OGD and EUS, only for non-complete clinical responders, to exclude disseminated disease
[9] PET-CT: during CRE-II and surveillance examinations, prior to OGD and EUS, for all patients (all were complete clinical responders during CRE-I) to guide OGD and EUS in targeting suspected locoregional laesions and to exclude disseminated disease
[10] Before inclusion, i.e. before **any** trial related procedure commences
[11] Only for patients with locoregional disease
[12] After CRE-II: Only for patients with cCR who are allocated to surgery
[13] Only for patients in whom a locoregional regrowth is highly suspected or proven, without any signs of distant dissemination
[14] After CRE-II: Only for patients with cCR
[15] Or when symptoms or results of any diagnostic test require shorter assessment intervals
[16] Quality of life will be assessed during the first 2 years only. Patients will be offered the possibility to summarize the quality of life outcomes in a logbook provided in the outpatient clinic. Patients will be asked to bring their personal logbook to the control appointments with the surgeon.
[17] Additional Blood (in Erasmus Medical Centre): during oncological check-up, additional blood will be drawn for the detection of circulating tumour-DNA. The maximum amount of additional blood that will be drawn for the detection of circulating tumour-DNA will be 200mL in 5,5 years (15mL per time point, so 13 time points).
[18] For patients allocated to surgery, a PET-CT will be performed at 16 and 30 months after completion of nCRT.

4. STUDY POPULATION

4.1 Study population

Operable patients with potentially curable locally advanced squamous cell- or adenocarcinoma of the oesophagus or oesophago-gastric junction.

4.2 Inclusion criteria

- Patients who underwent or are planned to undergo neoadjuvant chemoradiotherapy according to CROSS and are planned to undergo potentially curative surgical resection for histologically proven oesophageal or junctional squamous cell carcinoma or adenocarcinoma are eligible. Whenever pathology is inconclusive but a multidisciplinary expert group concludes oesophageal carcinoma because of radiologically or endosonographically highly suspected lesions, patients are eligible for the study.
- Age ≥18;
- Written, voluntary, informed consent.

4.3 Exclusion criteria

- Language difficulty, dementia or altered mental status prohibiting the understanding and giving of informed consent and to complete quality of life questionnaires;
- Non-FDG-avid tumour at baseline PET-CT scan;
- Initial treatment with endoscopic resection.

NB 'No-pass' on initial endoscopic ultrasonographic staging is not an exclusion criterion.

4.4 Sample size calculation

In the present phase III study, we plan to randomise at institutional level 280 patients with cCR during CRE-II between active surveillance and standard surgical resection. Simulation of trial outcomes with equal 2-year overall survival rates of 75% in both trial arms and an intra-correlation coefficient of 0.02 to account for between-institution variation (inter-quartile range for 2-year overall survival rates of 71% - 79) indicates a total sample size of 224 patients to show non-inferiority of surveillance to standard surgery with 80% power.(51) Non-inferiority is defined as a 2-year survival rate that is no more than 15 percentage points below the expected 75% 2-year survival rate among patients in the standard surgery group (data based on recent new data on clinically complete responders undergoing immediate surgery).(52)To allow for a 20% drop-out (*e.g.* patients in the active surveillance-arm who request immediate surgery in the absence of clinically proven or suspected regrowth) 280 patients are required for randomisation. Currently, the clinically complete response rate of all included patients is 34% in the SANO-trial during CRE-II, leading to a total required inclusion of 824 patients.

To reduce the number of newly included patients and to optimally use the data from the preSANO-trial, all recently (≥ May 2015) included patients with cCR during CRE-II from the current preSANO-trial who underwent bite-on-bite biopsies during CRE-I and CRE-II will be included in the control arm (see Figure 6 of the study protocol, n=29 patients, ethical approval will be requested). Assuming a 34% cCR rate*, the total number of required patients to be newly included in the SANO-trial will drop from 824 to 738patients. Consequently, patients with cCR are randomised at institutional level in a 4:5 ratio.
No interim analyses are planned for survival outcomes.

*Due to the enhanced insights in the efficacy of CREs, the rate of clinically complete responders can vary. Interim analysed will be performed frequently to keep the cCR rates up-to-date

Figure 5 - **Schematic overview of inclusion of clinically complete responders from preSANO-trial to reduce the number of newly included patients. See figure 10 for details on time periods, institutions and patient numbers.**

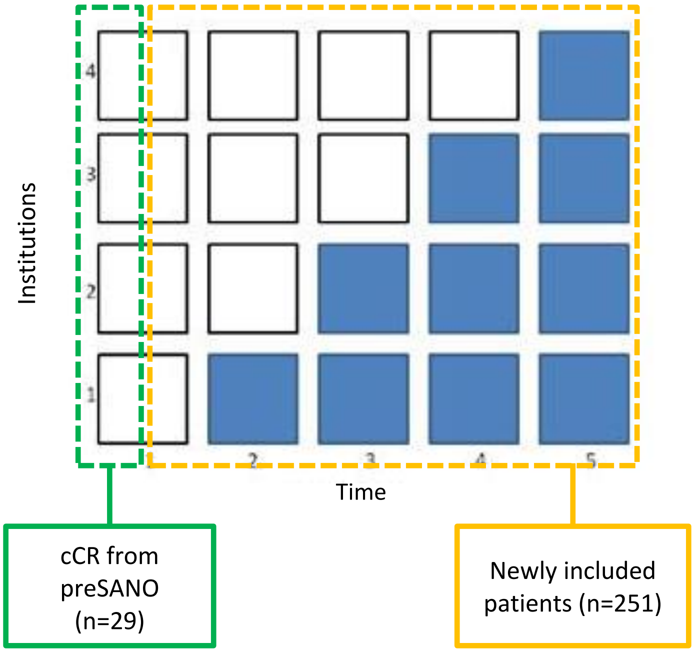

cCR: clinically complete response; white boxes: control group (standard surgery); blue boxes: intervention group (active surveillance)

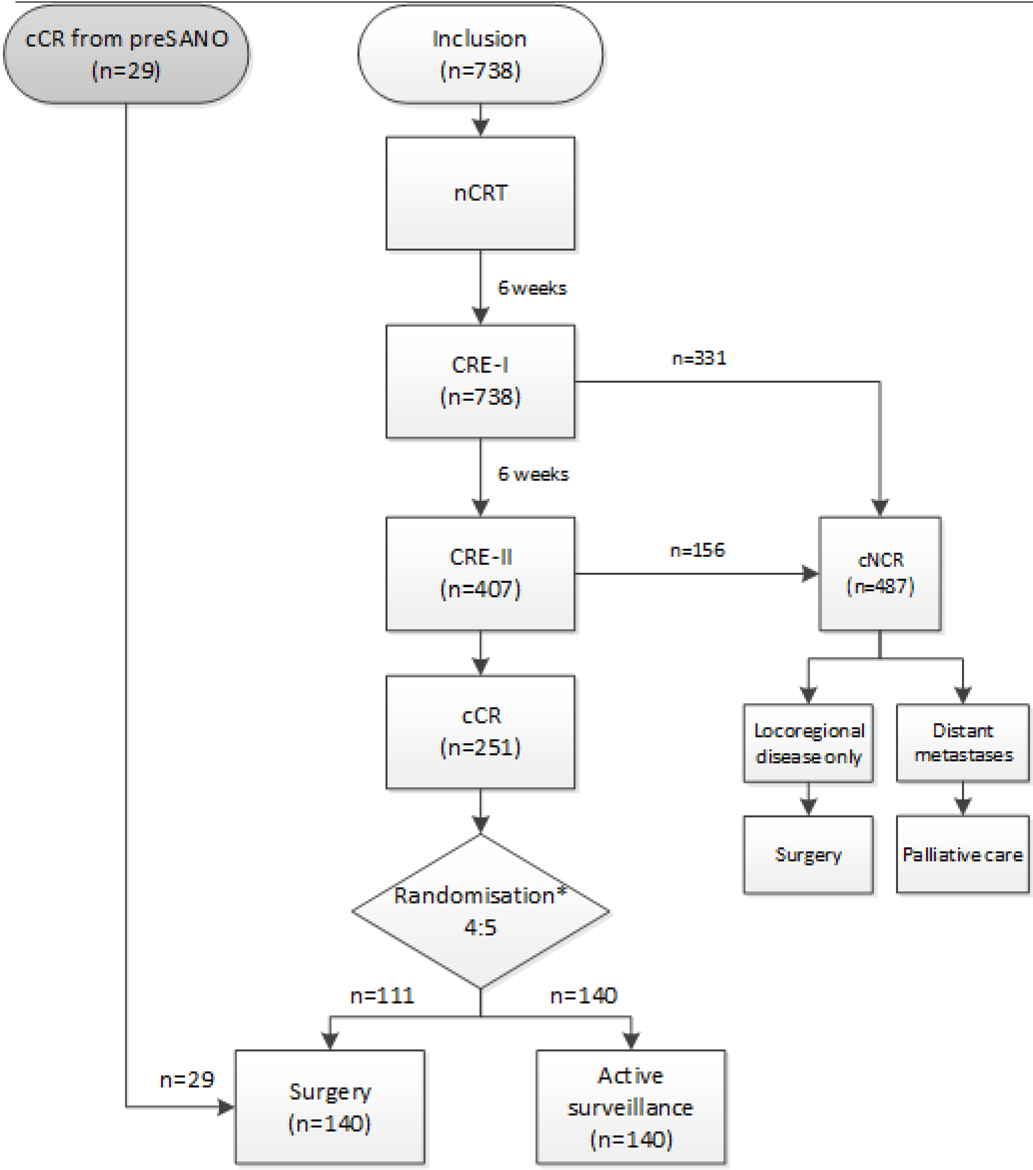

nCRT: neoadjuvant chemoradiotherapy; CRE: clinical response evaluation; cNCR: clinically non-complete response; cCR: clinically complete response. *At this point the patient will be allocated to one of the two treatment arms, dependent on the institution in which the actual treatment takes place. Randomisation has already been performed at institutional level (see §3.1 and §8.2) and will be known already at the moment of inclusion.

4.5 Expected recruitment per hospital in 3-year period (provisional)

Erasmus MC	Rotterdam	198
Catharina Cancer Centre	Eindhoven	60
Zuyderland Medical Centre	Heerlen	50
Radboud UMC	Nijmegen	60
Elisabeth Tweesteden Hospital	Tilburg	30
Gelre Hospital	Apeldoorn	30
LUMC	Leiden	30
Maasstad Hospital	Rotterdam	60
ZorgGroep Twente	Almelo	60
Netherlands Cancer Institute	Amsterdam	50
Reinier de Graaf Group	Delft	60
Medical Centre Leeuwarden	Leeuwarden	50
		738

5. TREATMENT OF SUBJECTS

5.1 Investigational product/treatment

5.1.1 Oesophagogastroduodenoscopy (OGD) and endoscopic ultrasonography (EUS)

Work-up at primary diagnosis

OGD

During OGD, locations of upper and lower tumour boundary are assessed. Also the upper oesophageal sphincter, Z-line (where the squamous epithelium of the oesophagus meets the columnar epithelium), oesophagogastric junction (upper border of gastric folds) and diaphragmatic impression (all given as the distance from the incisors in cm) are recorded. Photographic recordings are made of suspected laesions for future reference. If primary staging was done in a referring centre and not all of the previous data was noted, participating centres that are offering active surveillance have the choice to repeat the baseline OGD for accurate localisation of the primary tumour.

Radial EUS

Suspected locoregional lymph nodes, located at the paratracheal, aortopulmonary window, subcarinal, paraoesophageal, lesser curvature (= paracardiac + left gastric) and coeliac trunk stations (= coeliac trunk + common hepatic + splenic artery stations) will be counted and assessed for size, shape, echogenicity and border characteristics. Suspected laesion(s) in the left liver lobe and presence of ascites will be assessed. All findings will be registered in a standard diagnostic evaluation form.

CRE-I (4-6 weeks after completion of neoadjuvant chemoradiotherapy)

OGD

During OGD, photographic recordings will be made of the original primary tumour location and of suspected laesions for future reference. Video recordings will be made during the retraction of the endoscope. At least 8 biopsies, (4 bite-on-bite biopsies) will be taken of the most suspected laesions (Figure 9). In case of a post-chemoradiotherapy ulcer or –erosion, bite-on-bite biopsies will be taken at the borders of the ulcer (normal appearing mucosa combined with ulcerative tissue). If no laesions are seen, the original tumour location will be randomly biopsied using at least 4 bite-on-bite biopsies. Bite-on-bite biopsies are believed to have a better chance of detecting submucosal residual tumour than conventional biopsies (Figure 9). In case a severe stricture ("no-pass") results in a no-pass using the regular Q-endoscope, patients will be assumed to be 'disease positive' and referred for direct surgery. Even when biopsies can be obtained using the paediatric XP-scope, patient will still be assumed to be 'disease positive' due to the smaller biopsies taken with a paediatric XP-scope.

NB During CRE-I no EUS/FNA will be performed.

Figure 9 - **Bite-on-bite biopsies (1+2+3+4) supposedly increase the chance of detecting submucosal tumour deposits compared to conventional biopsies (1+2)**

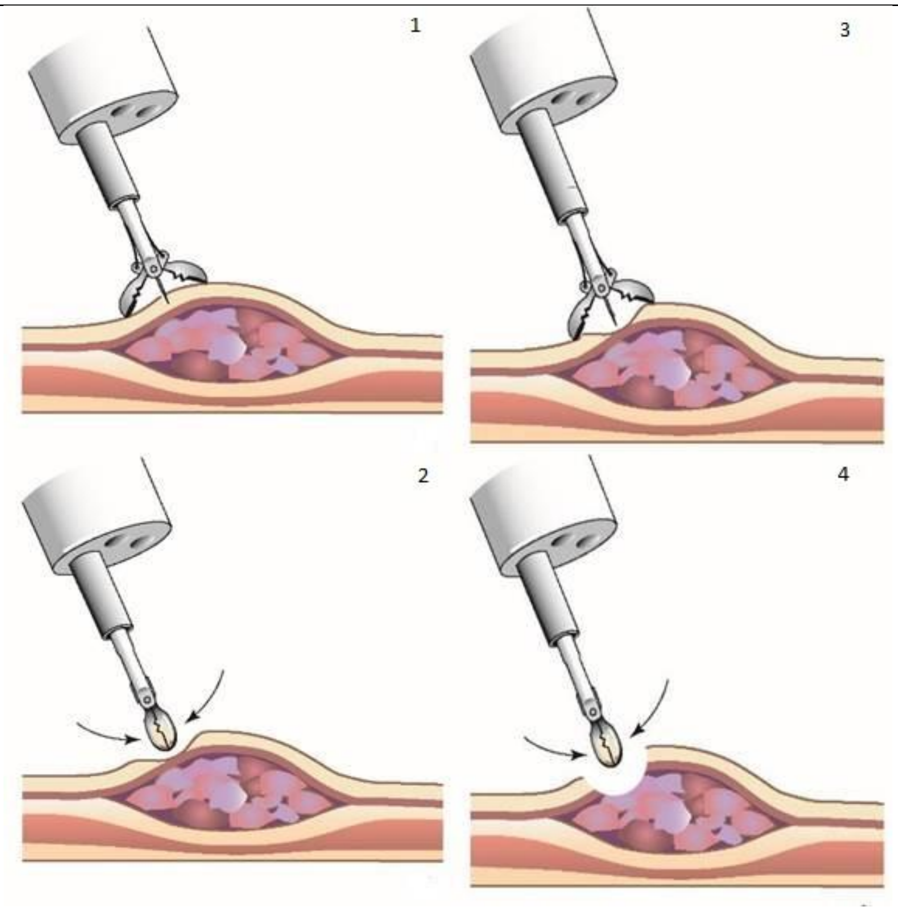

CRE-II (6-8 weeks after CRE-I; approximately 3 months after completion of neoadjuvant chemoradiotherapy) and all surveillance evaluations during follow-up (6, 9, 12, 16, 20, 24, 30, 36, 48 and 60 months after completion of neoadjuvant chemoradiotherapy)

<u>OGD</u>

During CRE-II and subsequent surveillance evaluations in the active surveillance arm 18F-FDG PET-CT will be performed prior to OGD. Any suspicious PET-positive laesions will be sampled, either by bite-on-bite biopsy during OGD or by FNA during subsequent linear EUS (see below). During CRE-II, at least 4 bite-on-bite biopsies will be taken of the most suspected laesions.

<u>Linear EUS</u>

During linear EUS, FNA will be performed of any suspected lymph nodes and / or other suspected laesions, <u>even if these lymph nodes are located directly behind the primary tumour site</u>.*,** In the case that non-representative material is obtained during FNA of suspected locoregional lymph nodes as determined by EUS and/or PET-CT during CRE-II or surveillance examinations, a second EUS+FNA will be scheduled <2 weeks. If during this second EUS+FNA, FNA is non-representative again, the patient will be classified as having a local regrowth and will be referred for surgery if no distant metastases are detectable. In case of a severe stricture (“no-pass”) with EUS during CRE-II, representative FNAs will not be available and therefore patients will be

assumed to be 'disease positive' and referred for surgery if no distant metastases are detectable. If suspected lymph nodes cannot be reached using FNA, patients will be considered disease positive.

All findings from CRE-I and CRE-II and all surveillance evaluations will be registered on a standard diagnostic evaluation form.

In case of a severe stricture ("no-pass") at CRE-I and/or CRE-II one should refrain from endoscopic dilation to minimise the risk of perforation.

* Cytology using FNA will also be obtained from lymph nodes that are located directly behind the primary tumour site, because the purpose of CRE-II is to detect any residual tumour, regardless of whether it is located at the primary tumour site or in the regional lymph nodes. Consequently, contamination is not an issue.
** In case of obvious macroscopic residual tumour during endoscopy, the gastroenterologist can decide to refrain from performing FNA of suspected locoregional lymph nodes during that endoscopic session. However, bite-on-bite biopsies should always be taken and if bite-on-bite biopsies turn out to be negative for residual tumour and no FNA of suspected lymph nodes was performed, a second EUS with FNA will be scheduled within 2 weeks, in order to sample any suspected lymph node.

5.1.2 PET-CT

All 18F-FDG PET-CT scans will be performed according to the EANM guidelines (53). Modern equipment, including multislice CT (16-slice or better) and if possible time-of-flight (TOF) PET, should be used. PET-CT scanners must be calibrated for (semi)quantitative measurements, according to Nedpas and/or EARL qualifications. Patients included in this study must have their follow-up PET-CT scans done on the same or identical type of scanner, under strictly the same conditions as their baseline PET-CT scan.

Briefly, key parameters are as follows:

- Adequate preparation (> 6 hrs. fasting), hydration (> 2 litre water) and control of diabetes mellitus before injection, and resting conditions after injection, according to local protocols and EANM guidelines. Patient must void before start of the PET-CT scan.
- Measurement of height, weight and blood glucose before injection.
- Injected activity of 18F-FDG in combination with the imaging time per bed position should be at least according to the EANM guidelines. For example, in Erasmus MC the activity for a 70kg patient is 220Mq of 18F-FDG, with 3min/bed acquisition. The total radiation dose of a PET-CT scan is approximately 4 mSv.
- The time interval between the injection and the actual start of the PET acquisition should be 60 min ± 5 min. Time of injection and start of PET must be recorded to the nearest min. Any reason for non-compliance to this criterion (if applicable) must be recorded.

- During scanning patients lie supine with their arms raised, if possible. The “total body” scan range must include skull base to upper thighs, with the low-dose CT in craniocaudal direction and PET acquisition in caudocranial direction.
- Processing and reconstruction: semi-quantitative evaluations (using SUV-max) should be performed on processed images without resolution-recovery and edge-enhancement techniques, according to EARL and Nedpas accreditation parameters. For visual assessment a separate, optimally processed set of images can be used.
- Quantification: SUV-max measurements. Additional SUV calculations with correction for blood glucose and body surface area may be performed later. SUV-peak measurements are not generally available on current software platforms, but will be considered at central final evaluation.

Complications related to all these diagnostic procedures will be monitored and recorded on a separate outcomes evaluation form, according to the definitions as described by the Esophagectomy Complications Consensus Group (ECCG).(54)

5.2 **Use of co-intervention (if applicable) NA**

5.3 **Escape medication (if applicable) NA**

6. INVESTIGATIONAL PRODUCT

6.1 **Name and description of investigational product(s) NA**

6.2 **Summary of findings from non-clinical studies NA**

6.3 **Summary of findings from clinical studies NA**

6.4 **Summary of known and potential risks and benefits NA**

6.5 **Description and justification of route of administration and dosage NA**

6.6 **Dosages, dosage modifications and method of administration NA**

6.7 **Preparation and labelling of Investigational Medicinal Product NA**

6.8 **Drug accountability NA**

7. NON-INVESTIGATIONAL PRODUCT

7.1 **Name and description of non-investigational product(s) NA**

7.2 **Summary of findings from non-clinical studies NA**

7.3 **Summary of findings from clinical studies NA**

7.4 **Summary of known and potential risks and benefits NA**

7.5 **Description and justification of route of administration and dosage NA**

7.6 **Dosages, dosage modifications and method of administration:**

7.7 **Preparation and labelling of Non Investigational Medicinal Product NA**

7.8 **Drug accountability NA**

8. METHODS

8.1 Study parameters/endpoints

8.1.1. Main study parameter/endpoint

Overall survival of patients with cCR at CRE-II (i.e. 12-14 weeks after completion of neoadjuvantchemoradiotherapy) for squamous cell- or adenocarcinoma of the oesophagus or oesophago-gastric junction.

8.1.2. Secondary study parameters/endpoints

- Percentage of patients in the active surveillance arm who do not undergo surgery (i.e. patients who are cured by neoadjuvant chemoradiotherapy or who have occult distant metastases during initial staging, which become manifest during active surveillance);
- quality of life as measured with EQ-5D (55), QLQ-C30 (56), QLC-OG25 (57) and Cancer Worry Scale (58) questionnaires;
- clinical irresectability (cT4b) rate;
- R0-resection rates defined as percentage of patients within the entire randomised group who undergo resection, defined as a tumour-free resection margin (margin >1mm not required, see also §8.7);
- postoperative morbidity/complications for all randomised patients with cCR who undergo resection, as defined by the ECCG (54);
- postoperative mortality for all patients with cCR who undergo resection, defined as 90 day- or in-hospital mortality;
- Progression-free survival, defined as the interval between randomisation and the earliest occurrence of disease progression resulting in primary (or peroperative) irresectability of disease, locoregional regrowth (after completion of therapy), distant dissemination (during or after completion of treatment);
- distant dissemination rate;
- quality adjusted life years (QALY, based on EQ-5D);
- cost-effectiveness.

8.2 Randomisation, blinding and treatment allocation

Phase III multi-centre stepped-wedge cluster randomised controlled trial.

RCTs randomising between surgical and non-surgical therapies on patient level often fail due to low inclusion rates. (45-48) Therefore, a stepped-wedge cluster design is preferred in the present trial. This design involves sequential crossover of clusters of participating institutions from control (standard surgery) to intervention (active surveillance). Randomisation is performed at institutional level, instead of at patient level (Figures 3 and 10). (50)

In the present trial, based on 12 participating centres (see §4.5), 6 clusters with comparable estimated inclusion rates will be formed, each cluster comprising 2 participating centres (preferably 1 academic centre and 1 non-academic teaching hospital). Based on the expected inclusion period of 36 months (see §3.2) and the inclusion of 29 clinically complete responders from the preSANO trial (see §4.4), every 3 months one cluster will cross over from control (immediate surgery) to intervention (active surveillance). Clusters will be determined by randomisation, but always consist of a centre with high and low expected total inclusion.

During the start-up phase of the trial, all centres will provide immediate surgery and will gain experience in the performance of response (and thus, surveillance) evaluations in at least 5 patients. When most centres are actively including patients, centres will be randomised to active surveillance approximately every 3 months. In order to insure optimal conditions and maximal safety for introducing the novel strategy (active surveillance), a cluster of 2 centres (Erasmus MC and either Zuyderland Medical Centre or Catharina Cancer Centre) with extensive experience in response evaluations and a large number of patients included in the preSANO-trial, will start to provide the intervention (active surveillance) after 3 months. After the next 3 months, another cluster of 2 participating centres (that included at least 5 patients) will be randomly assigned by the sponsor using a computer generated number sequence to begin with active surveillance and this procedure will be repeated after 3 months until all clusters have crossed over into the active surveillance group. The final phase of the trial, when all sites are participating in the intervention arm, finishes 6 months after the final 2 sites begin providing the intervention (Figure 10).

Expected numbers of patients included in both treatment arms during the different time periods and predefined clusters with comparable expected included patients are shown in Fig. 10. Inclusion rate will be closely monitored during the trial, and time periods can be adjusted if the number of included patients will differ substantially from the expectations.

The present stepped wedge design allows for control of underlying time trends, but reduces the period in which both control and experimental treatments are offered simultaneously, by providing immediate surgery in all centres during the start-up phase and by extending the final time period in which all centres are providing active surveillance. This will reduce the possible effect of selection bias due to patients' preferences in the period in which both control and experimental treatment are provided simultaneously.

After inclusion has been open for at least one year in all participating centres (*i.e.* one year after that date of local approval in the final centre), total inclusion will be monitored and a decision about continuation of the trial will be made in consultation with subsidizing parties KWF and ZonMw.

Figure 10 - **Stepped-wedge cluster design with addition of preSANO cCR-patients and sequential cross-over of 6 clusters comprising 2 centres approximately every 3 months.**

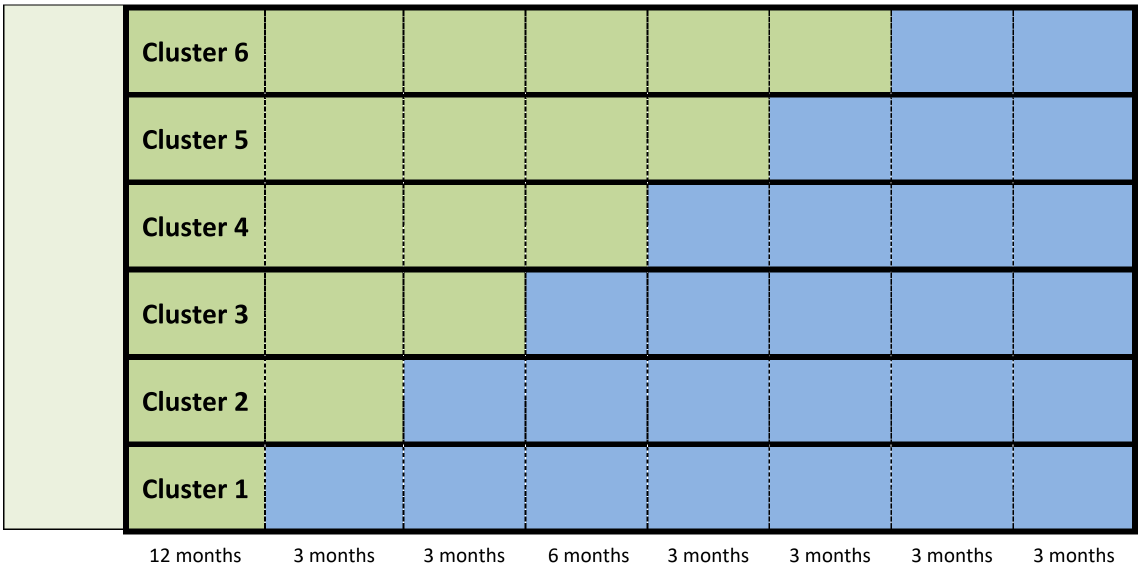

Expected number of patients with cCR included in each treatment arm per time period *

	29	54	18	14	22	2	1			N = 140
			9	14	32	24	27	16	16	N = 138
**Total**	**29**	**55**	**27**	**28**	**55**	**27**	**27**	**16**	**16**	**N = 280**

cCR from preSANO-2 (control)
control group (standard surgery)
intervention group (active surveillance)

cCR: clinically complete response (based on results from the SANO trial, it is expected that 34% of all included patients have a cCR).

* The first cluster has been extended until most of the centres were including patients. From that moment on, approximately every 3 months, 2 centres are randomised to the active surveillance arm.

**Predefined clusters***	**Expected total inclusion****
Erasmus MC (60) and Zuyderland Medical Centre (30) or Catharina cancer centre (45)	230-250
To be determined by randomization	80-120
To be determined by randomization	80-120
To be determined by randomization	80-120
To be determined by randomization	80-120
To be determined by randomization	80-120
**Total**	**738**

* Clusters will be randomly assigned by the sponsor using a computer generated number sequence to begin with active surveillance and this procedure will be repeated after roughly 3 months until all clusters have crossed over into the active surveillance group

** Expected total inclusion is defined as the total number patients who are included in the trial before the start of neoadjuvant chemoradiotherapy, regardless of the outcome of clinical response evaluations.

8.3 Study procedures

Patients will be included before the start, or shortly after completion, of neoadjuvant chemoradiotherapy. After written, voluntary informed consent and inclusion, patients will undergo neoadjuvant chemoradiotherapy according to the CROSS-protocol. Patients will be re-evaluated once or twice before undergoing surgical resection (in case of proven or highly suspected residual disease) or randomisation to one of the treatment arms (in case of a clinically complete response after both evaluations). These clinical response evaluations (CRE-I and CRE-II, see Figure 1) will consist of PET-CT (standard for all patients at CRE-II and only for positive patients at CRE-I), plus oesophagogastroduodenoscopy (OGD) with registration of endoscopic images for future reference and at least 4 bite-on-bite biopsies of the most suspected laesions, including (random) bite-on-bite biopsies at the site of the primary tumour, radial endoscopic ultrasonography (EUS) and linear EUS plus FNA of suspected lymph nodes (EUS only during CRE-II). The aim of both CREs will be to identify, by (cyto)histology, patients with residual and/or disseminated disease.

Patients with a clinically complete response after CRE-II will be randomised at institutional level between active surveillance and surgery (see §8.2). Patients in the active surveillance-arm will undergo PET-CT, OGD with at least 4 bite-on-bite biopsies will be taken of the most suspected laesions bite-on-bite biopsies and EUS (+/- FNA) every 3 months after the completion of neoadjuvant chemoradiotherapy in the first year, every 4 months in the second year, every 6 months in de third year and yearly in the 4th and 5th year of follow up, or when symptoms or results of any diagnostic test require shorter assessment intervals.
Surgical resection will be offered only to patients, in whom a locoregional regrowth is highly suspected or proven, without any signs of distant dissemination. Patients in the standard surgery arm will be offered surgery immediately after CRE-II (i.e. 12-14 weeks after completion of neoadjuvant chemoradiotherapy). In order to accurately compare distant dissemination rate between both treatment arms, an 18F-FDG PET-CT scan will be performed in all patients in the standard surgery arm 16 and 30 months after completion of neoadjuvant chemoradiotherapy .

CRE-I

The first CRE (CRE-I) is performed 4-6 weeks after completion of chemoradiotherapy (Figure 1). Patients with histological evidence of locoregional residual disease, but without evidence of disseminated disease, are offered immediate surgical resection. These patients have no benefit from postponement of surgery and should therefore have no delay in line with current practice. If the presence of locoregional residual disease is uncertain after CRE-1, surgical resection will be postponed for an additional 6 weeks.
Patients without histological evidence of locoregional residual disease and without disseminated disease are considered to be *clinically complete responders* and will be offered a postponed surgical resection. In these patients a surgical resection will be postponed for an additional 6-8 weeks, allowing patients more time to reach an optimal condition for surgery. Moreover, during several weeks immediately following neoadjuvant chemoradiotherapy a falsely positive signal is frequently detected by PET due to radiotherapy-induced inflammation and tumour necrosis. Based on what we already know from other malignancies, such as lymphoma and breast cancer (53, 59), PET-CT should be better able to guide targeted endoscopic and endosonographic biopsies at 12 weeks after nCRT. During this period, patients will be in contact with their

nurse practitioner, who will coordinate optimal supportive care, such as dietary supplementations and physical therapy/exercise (so called preconditioning).

CRE-II and surveillance evaluations

In the week preceding the planned postponed surgical resection (*i.e.* 11 weeks after completion of nCRT) a second clinical response evaluation (CRE-II) will be scheduled. CRE-II will be performed only in patients who were considered to be clinically complete responders (*i.e.* no viable tumour found) at CRE-I. The rationale to include a second clinical response evaluation before a planned surgical resection is to allow for a second chance at detecting residual- and/or disseminated disease. It is expected that during CRE-II (due to an extended time period from the end of neoadjuvant chemoradiotherapy) the 18F-FDG PET-CT signal will have a more favourable signal-to-noise ratio, because after 12 weeks the artefacts due to radiation-induced inflammation are expected to have largely dissolved.

All patients with cCR during CRE-II assigned to the active surveillance arm will undergo repeated surveillance evaluations similar to CRE-II every 3 months in the first year after completion of neoadjuvant therapy, every 4 months in the second year, every 6 months in de third year and yearly in the 4th and 5th year of follow up, or when symptoms or results of any diagnostic test require shorter assessment intervals.

Quality of life assessment

The Quality of Life(QoL) of the patients enrolled in this trial will be evaluated since active surveillance and avoidance of major surgery may lead to both short- and long-term effects on different aspects of QoL. To evaluate the well-being of patients two of the European Organization for Research and Treatment of Cancer Quality of Life questionnaires (EORTC QoL) will be used. The EORTC QLQ-C30 will be used to assess cancer specific QoL and the EORTC QLQ-OG25 will be used to assess tumour-specific QoL. Furthermore, the EQ-5D questionnaire will be used to facilitate calculation of quality adjusted life years. Finally, the Cancer Worry Scale will be used to investigate fear of residual disease and recurrence.

As part of the study protocol, patients will be asked to fill in QoL forms at the time of inclusion (baseline), at CRE-II (*i.e.* 3 months after completion of neoadjuvant chemoradiotherapy) and 6, 9, 12, 16, 20 and 24 months after completion of neoadjuvant chemoradiotherapy.

The SANO-trial has recently started a cooperation with the prospective observational cohort study of oesophagogastric cancer patients (POCOP) study. The POCOP-study monitors the QoL of oesophagogastric cancer patients with help of similar questionnaires used during the SANO-trial. In this cooperation, the POCOP study-coordinator will be responsible for the distribution of the QoL-questionnaires for patients participating both in SANO and in POCOP. Subsequent to the SANO-trial, patients will be asked if they give permission to receive the QoL questionnaires from the study-coordinator of the POCOP-study. If so, patients will be asked to give permission to share their contact details with the study-coordinator of the POCOP-study. If patients agree, their contact details will be given to the study-coordinator of the POCOP-study and patients will be contacted and informed about the POCOP-study (Figure 11). After informed

consent has been obtained, QoL-questionnaires will be distributed by the investigators of the POCOP-study according to the schedule of assessments as described above.

If patients do not give permission for inclusion in the POCOP-study, the baseline QoL form will be handed out to the patient by the local investigator or research nurse, after written informed consent has been obtained. The patient will receive instructions about completion. They will be asked to complete the questionnaires as soon as possible and return them. The local investigator, coordinator or research nurse will send the completed QoL forms to the Clinical Trial Center in the Erasmus MC Cancer Institute. Further QoL forms will be handed out during postoperative follow-up visits or during surveillance evaluations according to the schedule of assessments described above. Patients in the active surveillance arm will be requested to bring the completed forms to the out-patient clinic, which they visit one week after the diagnostic evaluation and regularly after the operation. Here, the nurse practitioner will examine the completed QoL forms, which allows discussion of specific complaints based on patients' answers.

Figure 11: **Flowchart SANO/POCOP patients**

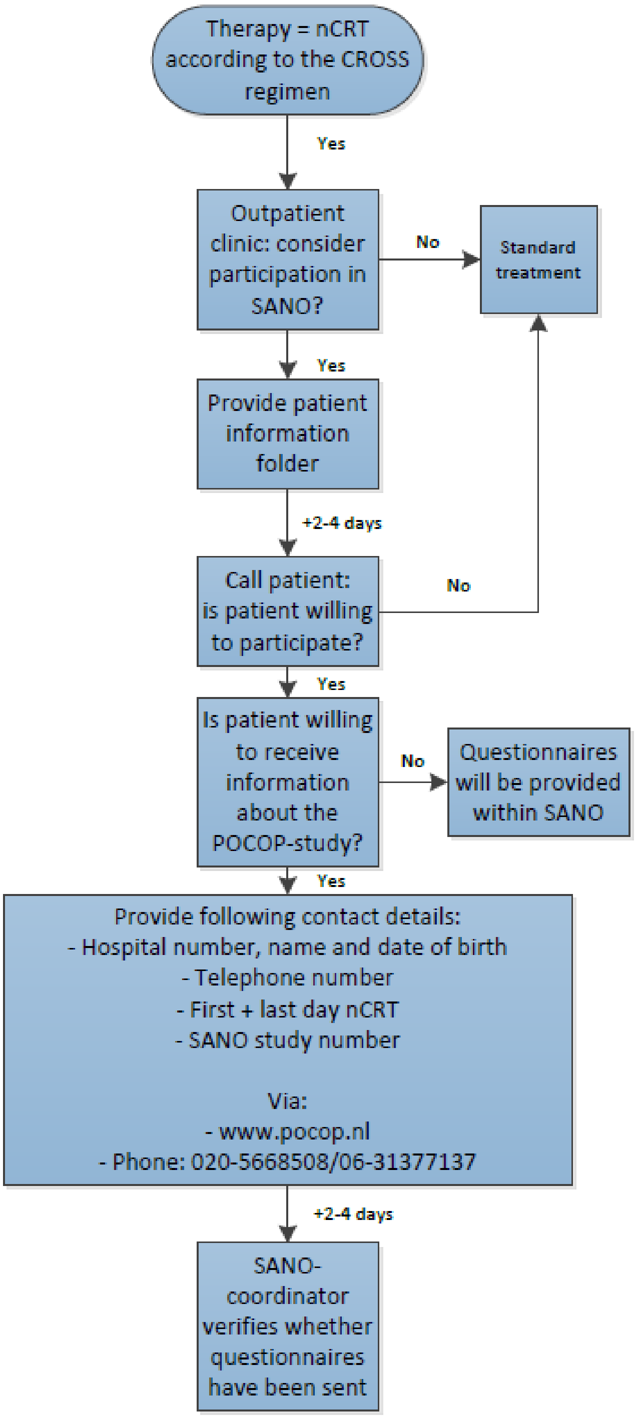

nCRT: neoadjuvant chemoradiotherapy, CROSS: chemoradiotherapy plus surgery versus surgery alone for oesophageal or junctional cancer, SANO: surgery as needed for oesophageal cancer, POCOP: prospective observational cohort study of oesophagogastric cancer patients.

Standard procedures (as part of standard treatment) within the study protocol:

8.3.1 Chemotherapy treatment
8.3.2 Radiotherapy treatment
8.3.3 Surgery
8.3.4 Pathology

8.3.1 Chemotherapy treatment

Paclitaxel 50 mg/m^2

Carboplatin AUC = 2

Administered by intravenous infusion on days 1, 8, 15, 22 and 29.

Premedication: all patients receiving Paclitaxel will receive premedication 30 minutes before the start of the Paclitaxel infusion according to the following recommended schedule:

Paclitaxel: Premedication		
Dexamethasone	10 mg iv	0.5 hour prior to Paclitaxel
Clemastine (Tavegil)	2 mg iv	0.5 hour prior to Paclitaxel
Ranitidine (Zantac)	50 mg iv	0.5 hour prior to Paclitaxel

At hour 0, the total calculated dose of Paclitaxel, diluted in 500 ml of normal saline will be infused over one hour. After the completion of the Paclitaxel infusion, 100 ml NaCl 0.9% will be infused over 0.5h, followed by an infusion of 8 mg Ondansetron (or its equivalent) diluted in 100 ml NaCl 0.9% over 0.5 hour. Hereafter the total calculated dose of Carboplatin, diluted in 500 ml glucose 5% will be infused over one hour (doses Carboplatin > 250 mg should be dissolved in 1000 ml glucose 5%). The absolute dose of Carboplatin will be calculated for the target AUC = 2 according to the following formula:

the absolute dose of Carboplatin = [target AUC] x (eGFR + 25).

formula GFR = [((140 – age) x 1.23 x body weight) / serum creatinin X (0.85 (female) or 1.00 (male))]

Paclitaxel/Carboplatin infusion scheme	
-0.5 hrs. start premedication	
0.0 hrs.	[…calculated dose…] Paclitaxel in NaCl 0.9% 500ml (PVC free)
1.0 hrs.	NaCl 0.9% 100 ml
1.5 hrs.	Ondansetron (or its equivalent) 8 mg in 100 ml NaCl 0.9%
2.0 hrs.	[…calculated dose…] Carboplatin in 500 ml glucose 5% in 1 hour

Patient monitoring

It is possible that some patients will experience asymptomatic bradycardia during the paclitaxel infusion. In addition, hypersensitivity reactions are possible and generally occur within the first few minutes of initiating the infusion. For these reasons, it is recommended that there is constant supervision and that the vital signs are monitored every fifteen minutes during paclitaxel administration. Thereafter, patients may be observed and heart rate and blood pressure checked if necessary, according to clinical symptoms.

8.3.2 Radiotherapy treatment

The radiotherapy protocol of the SANO-trial has been authorized by the platform for radiotherapy of gastro-intestinal tumours of the Dutch society for radiotherapy and oncology.

Fractionation schedule

A total dose of 41.4 Gy will be given in 23 fractions of 1.8 Gy, 5 fractions per week, starting at the day of the first cycle of chemotherapy. All patients will be radiated by external beam radiation.

Simulation procedure

Prior to the start of the irradiation a planning (PET-)CT will be made from the cricoid to L3 vertebra with a maximal slice thickness of 5 mm, with the patient in supine position. Reproducibility will be assisted by orthogonal laser beams and landmarks for the isocentre. The arms of the patient will be in abduction of more than 90 degrees, supported by devices to insure stability and reproducibility of the treatment set up.

Definitions of target volumes and critical structures

GTV: The Gross Tumour Volume is defined by the primary tumour (GTVp) and suspected enlarged regional lymph nodes (GTVn) and will be delineated on each relevant CT slice. The GTV will be determined using all available information (physical examination, endoscopy, EUS, CT of neck/thorax/abdomen and PET-CT).

CTV: The Clinical Target Volume is defined by the GTVn and GTVp plus the area of regional lymph nodes up to at least 3 cm in cranial and caudal extension of the oesophagus from the GTVp. To ensure adequate radial margins around the macroscopic primary tumour, a minimum radial CTV-GTVp margin of 3 mm into peritumoral fatty tissue is required. For distal tumours the caudal margin should follow the wall of oesophagus and cardia. The margin in the direction of the wall of the cardia can be limited to 2 cm. The area of regional lymph nodes is defined by the fatty peri-oesophageal tissue limited by the pleura in lateral direction, by the pericardium or large vessels in ventral direction and by the vertebra in dorsal direction. Paravertebrally the CTV extends up to the right sided azygos vein and to the midline of the aorta in case the aorta descends paravertebrally on the left side of the spine. Due to anatomical variations, it is possible to deviate from these anatomical landmarks as long as the fatty peri-esophageal tissue is included in the target volume. The CTV includes all the fatty tissue along the left gastric artery (between cardia and liver), of the aortic-pulmonary window, of the subcarinal, pretracheal and supraclavicular region as far as they are within the 3 cm craniocaudal range from the primary tumour. In case of pathological lymph nodes, the CTV is

extended up to the level of the pathologic nodes with a GTVn-CTV margin of 5mm. In case of discrepancies between diagnostic tools (EUS, CT scan or PET-CT) about the pathology of lymph nodes, the advice is to include them in the CTV if at least one of the diagnostic modalities suggests suspect nodes.
PTV: The Planning Target Volume (PTV) will consist of the CTV plus a margin of 1 cm in all directions for organ mobility and setup inaccuracy. In case of proximal or mid-oesophageal tumours the margin can be reduced to 7 mm in transversal directions if positional verification with cone beam CT scans or online MRI (MRI linac) are used. When breath hold techniques or adaptive margin techniques are used, or a MIP (maximum intensity projection) of a 4-D CT is used for delineation, it is allowed to reduce the margin for organ mobility and set up inaccuracy. Critical organs: both lungs and heart will be contoured on all slices. For heart delineation the heart atlas of Feng et al. is used (67) . The kidneys should be contoured if the lower border of the PTV extends up to the level of one third of one of the kidneys.

Radiation technique

The most appropriate technical solutions (e.g. beam quality, field arrangement, conformal therapy planning) will be chosen as long as they comply with the prescribed clinical goals, dose constraints, ICRU 83 (2010) safety margins and homogeneity requirements.

Normal tissue tolerance

Dose-volume histograms (DVH’s) of lungs, the heart and if applicable the kidneys will be obtained for all patients. DVH’s will be used to document the normal tissue damage and may help to select the most appropriate treatment plan. The aim is to minimise dose in normal tissues as much as possible.
The mean dose of both lungs should not exceed 16 Gy and V20 ≤ 30%. The heart volume receiving > 40 Gy should not exceed 30%. At least 2/3 of the volume of one normally functioning kidney should receive less than 18 Gy.

**Organ at risk**	**Maximally allowed dose**
heart	V40≤30%
kidneys	D67%<18 Gy (for one normally functioning kidney)
lungs	V20≤30%, mean lung dose ≤16Gy

Treatment planning, dose calculation and set up verification

The prescription dose will be specified at the ICRU 83 reference point, which will be the isocenter for most patients. The daily prescription dose will be 1.8 Gy at the ICRU reference point and the 95% isodose must encompass the entire planning target volume (PTV) for at least 95% of the volume (95% because of adjacent lung tissue; strive for >97%). The maximum dose in the PTV must not exceed the prescription dose by >7% (ICRU 83 guidelines).

Treatment verification

Position verification and correction during radiation should be done by cone beam CT or MRI verification according to the institutional protocol.

8.3.3 Surgery

Surgical resection will be attempted immediately after CRE-I only in those patients who present at CRE-I with histologically proven residual disease after completion of neoadjuvant chemoradiotherapy, without any signs of disseminated disease. All other patients will undergo surgical resection after CRE-II in the absence of distant metastases, unless randomised for active surveillance. In the active surveillance arm, surgical resection will be offered only to those patients, in whom a locoregional regrowth is highly suspected or proven, without any signs of distant dissemination.

For carcinomas that extend proximal to the inferior pulmonary vein a transthoracic oesophageal resection is preferred. For carcinomas that do not extend proximal to the inferior pulmonary vein, a transthoracic approach with a two-field lymph node dissection or a transhiatal approach can be performed, depending on both patient characteristics and local expertise. Both open, hybrid and completely minimally invasive techniques are allowed. At least 15 lymph nodes dissected should be aimed for in every patient. Minimal requirement for nodal resection in the chest should include subcarinal and para-oesophageal lymph node stations (see Appendix A). Lymph nodes should be stored in separate boxes per station. This will ensure more precise registration of lymphatic dissemination per lymph node station.

A wide local excision of the primary tumour and the regional lymph nodes is carried out including a standard dissection of the lymph nodes around the coeliac axis (separately collected for nodes along the left gastric, common hepatic and splenic artery). The continuity of the digestive tract will preferably be restored by a gastric tube reconstruction or if required by a colonic interposition.

8.3.4 Pathology

All CRE- and surveillance biopsies will be assessed by expert GI pathologists. Initially, all biopsies will be analysed based on the regular HE slide (which contains two or three levels). If analysis at these levels reveals obvious vital tumour, the biopsy will be classified (diagnosed) as positive. If the assessment of this HE slide is negative for malignancy (no malignancy), deeper sections will be performed (two or three additional levels, depending on the amount of tissue on the paraffin block). In case of doubt regarding the presence of tumour (cells) after analysis of a biopsy at the aforementioned additional levels, extra dPAS and (pan)keratin staining will be performed. In case of an originally diagnosed signet-ring cell carcinoma or a poorly cohesive carcinoma with mucin production, analysis at three additional (deeper) levels and dPAS and keratin staining will be performed consistently.

Only the CRE- and surveillance biopsies with uncertain outcome or with high-grade dysplasia will be revised at the Department of Pathology of the Erasmus MC or by a second pathologist in the participating centre following the same strategy, using a standard protocol. In case of a discordant result, the specimens will be reviewed by a third independent expert GI pathologist and a consensus diagnosis should be reached if at least two pathologists agree. In case the revision concludes high-grade dysplasia, the CRE will be considered positive. In case the results remain uncertain, a multidisciplinary expert group at the Erasmus MC will reach consensus on whether or not patients will be treated for oesophageal cancer, taken into account the condition of the patient and other diagnostic modalities like 18-FDG PET-CT.

The resection specimens will be assessed using the 7th edition of the UICC TNM cancer staging. Microscopically radical resection (R0) will be defined as a tumour-free resection margin (margin >1mm not required). Also, prepTNM staging will be estimated as described earlier. (60) In these resection specimens special attention will be given to the effects of the nCRT, i.e. tumour reduction and therapy effects. It might be difficult to recognise tumour tissue macroscopically as a consequence of the nCRT. The estimated location of the primary laesion (tumour-bed) plus surrounding areas need to be embedded in total in order to adequately judge residual tumour and therapy effects. Tumour regression grade (TRG) will be noted according to Mandard classification (TRG 1 to 4). TRG4 implies the finding of a vital tumour with no visible therapy effects while in TRG1 specimens, no vital tumour cells are detected after complete histological examination of the tumour area. In many cases a multifocal tumour appearance is present with intertwined therapy effects, usually recognized as fibrosis. Other features of therapy effects are necrosis, inflammation with multinucleated giant cells, mucin lakes and calcifications. In some cases only scattered tumour cells are visible, often with bizarre morphologies. In these cases a keratin stain could be performed to confirm the epithelial nature of the atypical cells.

The lymph node dissection should contain at least 15, but preferably 23 nodes derived from the mediastinum and upper abdomen, which are essential for correct ypTNM staging. The use of the PPMI PALGA protocol for oesophagus is recommended to assure complete and uniform registration of various pathology parameters.

8.4 Withdrawal of individual subjects

Subjects can leave the study at any time for any reason if they wish to do so without any consequences. The investigator can decide to withdraw a subject from the study for urgent medical reasons.

8.4.1 Specific criteria for withdrawal NA

8.5 Replacement of individual subjects after withdrawal

Subjects who withdraw from the trial will not be replaced. We have accounted for an expected (voluntary) withdrawal of approximately 12% of included patients in the power calculation.

Patients who withdraw due to cross-over between treatment arms (*i.e.* patients allocated to the standard surgery arm who refuse to undergo surgery and patients allocated to the active surveillance arm who request for immediate surgery) will be registered in the database to allow for per protocol comparative analysis.

8.6 Follow-up of subjects withdrawn from treatment

Follow-up of subjects withdrawn from treatment will be according to the standard follow-up protocol offered to patients during standard care.

8.7 Premature termination of the study

The safety and feasibility of this trial depend on several factors, namely:

1. timely detection of resectable locoregional regrowth (<T4b) in the active surveillance arm;
2. feasibility of achieving a radical resection in the active surveillance arm;
3. acceptable postoperative morbidity for delayed surgery in the active surveillance arm;
4. acceptable distant dissemination rate in the active surveillance arm (as compared to the immediate surgery arm).

Delaying surgical resection in patients in the active surveillance arm should neither lead to a significant decrease in tumour resectability and radical resection rate, nor to a significant increase in postoperative mortality and distant dissemination rate. We therefore incorporate a series of stop rules to be tested (early) during the course of the trial (see below). Each stop rule will be repeatedly tested when the first 10, 20, 30 and 50 events for that particular stop rule have occurred in the active surveillance arm (*i.e.* [ad 1 and 2] detection of locoregional regrowth, [ad 3] the performance of delayed surgery or [ad 4] the detection of distant metastases).
However, to correct for the possible rarity of regrowths and/or resections in the active surveillance arm (and to prevent the reaching of a stop rule due to under-sampling), stop rule percentages are defined as a proportion of all included patients into the active surveillance arm until the moment of the most recent event occurrence. For example: in the first 10 detected regrowths, 3 patients are found to have an irresectable regrowth. However, these 10 regrowths were detected after 30 patients were included into the active surveillance arm. Under these circumstances, the stop rule percentage would be 3 out of 30 (10%), and not 3 out of 10.

Stop rules

Stop rules	After 10 events	After 20 events	After 30 events	After 50 events
1. Proportion of all patients in the active surveillance arm that present with an irresectable or incurable (T4b or R2) regrowth, in the absence of distant metastases.[1]	≥ 14%	≥ 10%	≥ 8%	≥ 6%
2. Proportion of all patients in the active surveillance arm that undergo a microscopically non-radical (R1) resection.[2]	≥ 18%	≥ 15%	≥ 14%	≥ 12%
3. Postoperative morbidity:<br>- Postoperative in-hospital mortality in all patients in the active surveillance arm.[3]	≥ 13%	≥ 12%	≥ 10%	≥ 9%
- Proportion of all patients in the active	≥ 17%	≥ 13%	≥ 11%	≥ 10%

surveillance arm with hospital stay >60 days or who develop postoperative trachea-neo-oesophageal fistula.[4]	
4. Proportion of all patients in the active surveillance arm that develop distant dissemination after one and two years of follow-up.[6]	One- and two year distant dissemination rates[7] may not be significantly higher than in the standard surgery arm (analysis after 10, 20, 30 and 50 patients with distant dissemination in the active surveillance arm).

[1] Percentages are based on 4, 6, 7 and 8 irresectable regrowths in the active surveillance arm after 10, 20, 30 and 50 regrowths, resp., assuming regrowths in one third of all patients in the active surveillance arm.
[2] Percentages are based on the upper limit of the 95% confidence interval of 8% (R1 resections, based on CROSS-trial(18)) after 10, 20, 30 and 50 resections, resp., assuming regrowths in one third of all patients in the active surveillance arm.
[3] Numbers are based on the upper limit of the 95% confidence interval of 5% (30-day postoperative or in-hospital mortality) after 10, 20, 30 and 50 resections, resp., assuming regrowths in a third of all patients in the active surveillance arm.
[4] Percentages are based on the upper limit of the 95% confidence interval of 4% (hospital stay>60 days or trachea-neo-oesophageal fistula, based on Dutch Upper-GI Cancer Audit [DUCA] data, 2016) after 10, 20, 30 and 50 resections, resp., assuming regrowths in one third of all patients in the active surveillance arm.
[5] χ^2 test or Fisher's exact test will be used for comparison of both groups, statistical significance will be set at 0.05.
[6] Distant dissemination rate will be defined as the percentage of all included patients in a treatment arm with histologically proven or radiologically highly suspected distant dissemination.

Examples

Ad.1 If the first 10 regrowths in the active surveillance arm were detected after 30 patients were included into that arm, stop rule 1 is reached if ≥5 (≥ 14% of 30) of these patients present with an irresectable (T4b or R2) regrowth.

Ad.2 If the first 10 resections in the active surveillance arm were detected after 30 patients were included into that arm, stop rule 2 is reached if ≥6 (≥ 18% of 30) of these patients undergo a microscopically non-radical (R1) resection.

Ad.3 If the first 10 resections for disease regrowth in the active surveillance arm were performed after 30 patients were included into that arm, stop rule 4 is reached if ≥4 (≥ 13% of 30) of these patients die postoperatively within 30 days or in-hospital.

Ad.4 If the first 10 patients who develop distant metastases within one year of follow-up in the active surveillance arm were detected after 30 patients were included into that arm, stop rule 4 is reached if ≤3 out of 30 patients (p=0.03) in the control arm have developed distant metastases within one year of follow-up.

All stop rule parameters will be reported to the sponsor by the local investigator without undue delay after obtaining knowledge of the events. In case one of the stop rules is reached, all participating centres will be notified immediately and further inclusion will be stopped. Patients who have been already included will be informed and offered the possibility of immediate (high-priority) surgical resection, even in the absence of suspicion of regrowth. Continuation of surveillance will also still be offered. The final decision of how to proceed will be made by the patient together with the local multidisciplinary team after extensive deliberation. The accredited METC will be notified within 15 days, including the reasons for the premature termination of the trial.

8.8 Expected outcome active surveillance arm

Based on the available literature and preliminary data from the preSANO trial, we expect that 18% of all patients who underwent nCRT (*i.e.* 37% of all patients with cCR) will be cured without surgery after 5 years of active surveillance and that in 17% of all patients who underwent nCRT (*i.e.* 33% of all patients with cCR) distant metastases will become manifest during active surveillance. Most distant metastases are expected to be detected within 2 years of follow-up. Taken together, this will lead to avoidance of unnecessary surgery in 35% of all patients (*i.e.* 70% of all patients with cCR).

Furthermore, we expect that 13% of all included patients who underwent nCRT (*i.e.* 27% of all patients with cCR) will develop a resectable locoregional regrowth (*i.e.* <T4bM0).

Finally, we expect that 1.5% of all patients who underwent nCRT (*i.e.* 3% of all patients with cCR) will develop an irresectable locoregional regrowth without distant metastases (T4bM0). These patients will be referred for palliative care.

NB Based on the literature we do not expect novel distant metastases that develop from post-nCRT locoregional residual or recurrent disease.

	% of all included patients	% of patients with clinically complete response after CRE-II
Cured without surgery	18	37
Distant metastases (+/- locoregional regrowth)	17	33
Locoregional regrowth (<T4b) without distant metastases	13	27
Locoregional regrowth (>T4b)	2	3

Patients with benefit from active surveillance

Patients without benefit and without harm

Patients with harm from active surveillance

Figure 11 - **Patient distribution active surveillance-arm**

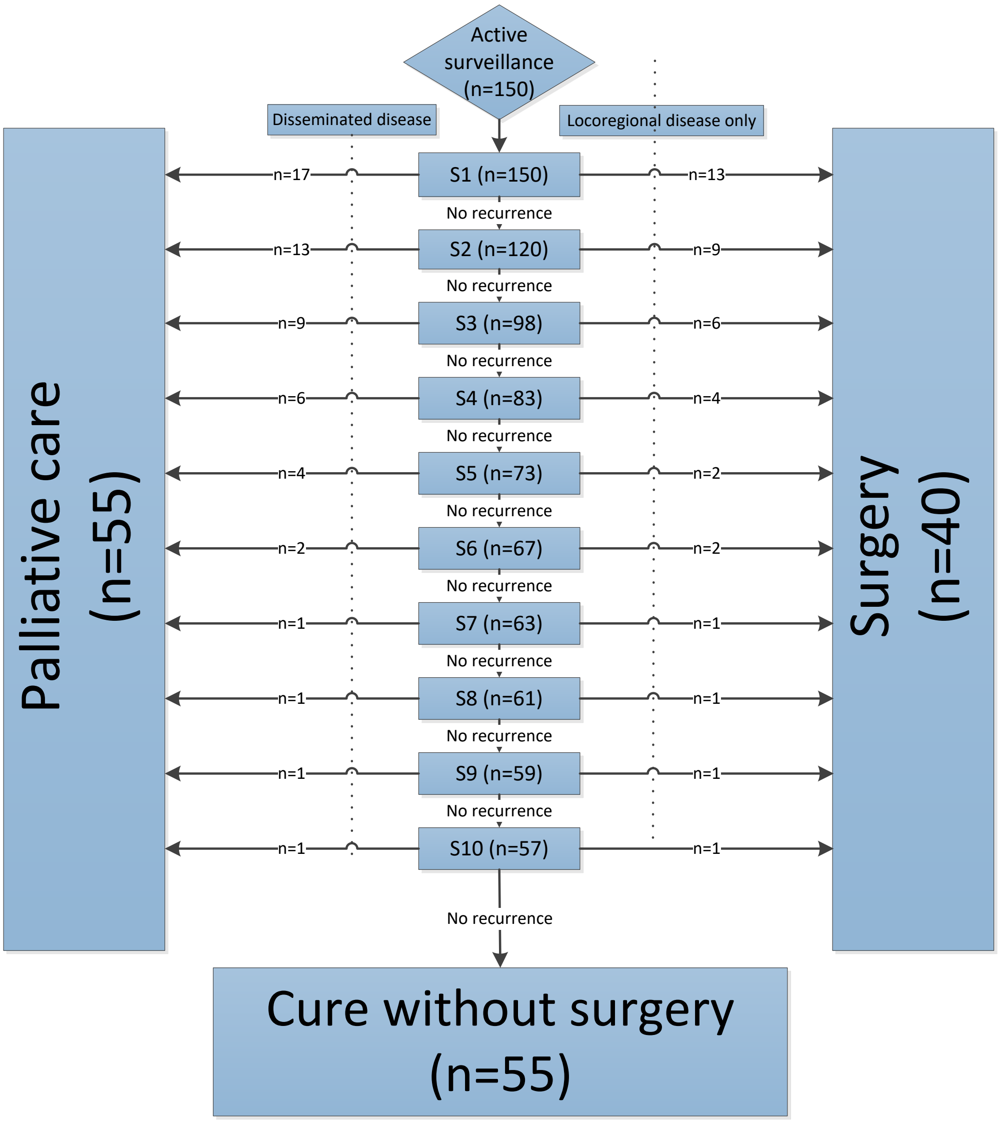

8.9 Proctoring

Gastroenterology

From each centre with limited experience with clinical response and surveillance evaluations, one or two gastroenterologists will participate in a training session, organized by a centre with more experience in response evaluation after CROSS chemoradiation.

Surgery

For all postponed resections in the active surveillance arm, on-site proctoring by an experienced upper-GI surgeon will be organized. Additionally, surgeons from participating centres with limited experience in delayed surgery will be invited to attend delayed surgical procedures in nearby experienced centres.

9. SAFETY REPORTING

9.1 Section 10 WMO event

In accordance to section 10, subsection 4, of the WMO, the sponsor will suspend the study if there is sufficient ground that continuation of the study will jeopardise subject health or safety. The sponsor will notify the accredited METC without undue delay of a temporary halt including the reason for such an action. The study will be suspended pending a further positive decision by the accredited METC. The investigator will take care that all subjects are kept informed.

9.2 AEs, SAEs and SUSARs

9.2.1 Adverse events (AEs)

Adverse events are defined as any undesirable experience occurring to a subject during the study, whether or not considered related to the diagnostic and therapeutic procedures. Only adverse events and complications related to experimental study procedures (delayed surgery, response evaluations and surveillance evaluations) throughout the trial reported spontaneously by the subject or observed by the investigator or his/her staff will be recorded within the trial (including postoperative complications). Adverse Events will be reported during the period of time from the day that the patient will undergo randomisation (at institutional level) until the end of follow-up.

9.2.2 Serious adverse events (SAEs)

A serious adverse event is any untoward medical occurrence or effect that occurs during the period of time from the day that the patient will undergo CRE-I until the day that the patient will undergo surgery, that :

- results in death;
- is life threatening (at the time of the event);
- requires hospitalisation or prolongation of existing inpatients' hospitalisation;
- results in persistent or significant disability or incapacity;
- any other important medical event that did not result in any of the outcomes listed above due to medical or surgical intervention but could have been based upon appropriate judgement by the investigator.

An elective hospital admission will not be considered as a serious adverse event.

The following events are not considered SAEs:

Events and complications unrelated to experimental study procedures (delayed surgery, response evaluations and surveillance evaluations) are not considered SAE's

The Local Investigator will decide whether or not the serious adverse event is related to any study related procedure. The assessment of causality is made by the local investigator using the following:

Relationship	Description
Unrelated	There is no evidence of any causal relationship
Related	There is (even only little) evidence to suggest a causal relationship

The local investigator will report all SAEs to the sponsor within 24 hours after obtaining knowledge of the event.

The sponsor will report the SAEs through the web portal *ToetsingOnline* to the accredited METC that approved the protocol, within 7 days of first knowledge for SAEs that result in death or are life threatening followed by a period of maximum of 8 days to complete the initial preliminary report. All other SAEs will be reported within a period of maximum 15 days after the sponsor has first knowledge of the serious adverse events.

9.2.3 Suspected unexpected serious adverse reactions (SUSARs) NA

9.2.4 Events of clinical interest

Events of clinical interest should also be reported expedited to the sponsor within 7 days after first knowledge by the Local Investigator.
These events comprise the following parameters (in both treatment arms), identified to define stopping rules:

1. The occurrence of irresectable or incurable (T4b or R2) regrowth in patients allocated to the surveillance arm
2. Microscopically non-radical (R1) resection in patients in the active surveillance arm
3. Postoperative in-hospital mortality in patients in the active surveillance arm.
4. The occurrence of distant dissemination in patients in the active surveillance arm during the first two years of follow-up

9.3 Annual safety report NA

9.4 Follow-up of adverse events

All AEs will be followed until they have abated, or until a stable situation has been reached. Depending on the event, follow-up may require additional tests or medical procedures as indicated, and/or referral to the general physician or a medical specialist.
SAEs need to be reported during the period of time from the day that the patient will undergo CRE-I until the end of follow-up, as defined in the protocol.

9.5 Safety committee

A safety committee will be established to perform on-going safety surveillance and to perform interim analyses to assess the safety data and the stop rules as described in §8.7. Each stop rule will be repeatedly tested when the first 10, 20, 30 and 50 events for that particular stop rule have occurred (*i.e.* [ad 1 and 2] detection of locoregional regrowth, [ad 3] the performance of delayed surgery or [ad 4] the detection of distant metastases).

The safety committee will consist of one surgeon (prof.dr. C. Verhoef) and one medical oncologist (dr. E. van Meerten) from the Erasmus MC, who are both unrelated to this study and have no conflict of interest with the coordinating investigator of the study. Also, prof. dr. E.W. Steyerberg, from the department of Public Health, will be consulted during interim analyses to advice on statistical uncertainty of complications and outcome measurements.

The safety committee will continuously review whether adverse events (AEs) as reported by the participating centres fulfil the criteria of 'serious complication' as defined in the stopping rules.

The safety committee will also continuously monitor whether any of the stopping rules have been reached (see 8.7 Premature termination of the study). If one of the stopping rules is reached the safety committee will notify the coordinating- and principal investigators, after which all participating centres will be notified immediately and further inclusion will be stopped. Patients already included will not undergo any further research-related tests and will be offered surgical resection (= standard treatment) with minimal additional delay. The accredited METC will be notified within 15 days, including the reasons for the premature termination.

9.6 Resonance group

To involve patient organisations during the performance of the trial, a resonance group will be established , consisting of representatives from each participating specialty (*i.e.* surgery, gastroenterology, nuclear medicine, medical oncology, radiotherapy, pathology and radiology), a representative from Stichting Patiënten Kanker Spijsverteringskanaal (SPKS) and a representative from Patiëntenfederatie Nederland.

This resonance group will organize meetings three times in the first year of the trial, twice in the second year, once in the third year, and once after completion of inclusion.

10. STATISTICS

10.1 Primary study parameter(s)

The difference in survival over a 2-year horizon between the control and the experimental treatment arm will be analysed with a mixed-effects Cox regression model. Use of a mixed regression model – including an institution-level random effect – is required to capture the potential between-institutional variation in survival [ref T M Therneau and P M Grambsch, Modeling Survival Data: Extending the Cox Model, Springer-Verlag, 2000]. To correct for potential selection bias, the treatment effect will be estimated with adjustment for prognostic factors for survival, i.e. age, sex, histologic subtype of tumour, clinical N stage, and WHO performance score. We will also use the mixed-effects Cox regression model to study potential differences in treatment effect between subgroups of patients. Subgroups are predefined according to age, sex, histologic subtype of tumour, clinical N stage, and WHO performance score.

10.2 Secondary study parameter(s)

- Quality of life data will be analysed according to the EuroQol, EORTC and Cancer Worry Scale scoring manuals. (40-43) Repeated measurement analysis will be used to evaluate within and between group differences;
- The percentage of patients who avoid potentially redundant surgery will be described (*i.e.* patients who are cured by neoadjuvant chemoradiotherapy or who have occult distant metastases during initial staging, which become manifest during active surveillance);
- The comparison of clinical irresectablity (cT4b) rates between both study arms will be analyzed by logistic regression analysis;
- The comparison of R0-resection rates in both study arms will be analyzed by logistic regression analysis;
- Distant dissemination rates will be analysed after one and two years of follow-up in both study arms and will be compared by logistic regression analysis;
- Postoperative morbidity and mortality, as defined in §8.1 of the study protocol, will be described; Progression-free survival, as defined in §8.1, will be analysed statistically similar to overall survival (see primary study parameter);
- Cost-effectiveness analysis: see WP 4.

10.3 Other study parameters NA

10.4 Interim analysis (if applicable)

Interim analyses will be performed to assess the stop rules as described in §8.7. Each stop rule will be repeatedly tested when the first 10, 20, 30 and 50 events for that particular stop rule have occurred (*i.e.* [ad

1 and 2] detection of locoregional regrowth, [ad 3] the performance of delayed surgery or [ad 4] the detection of distant metastases). In case a stop rule is reached, the trial will be stopped prematurely.

Furthermore, in order to inform the research community about results in an early phase, an interim analysis will be performed after a minimum follow-up of two years for all included patients.

11. ETHICAL CONSIDERATIONS

11.1 Regulation statement

The study will be conducted according to the principles of the Declaration of Helsinki (10th version, Fortaleza, 2013) and in accordance with the Medical Research Involving Human Subjects Act (WMO) and other guidelines, regulations and Acts.

11.2 Recruitment and consent

The supervising doctor or any other doctor of the multidisciplinary team will inform subjects about the study and ask for their consent. Patients will be informed about the stepped wedge design and both treatment arms, regardless which treatment arm is offered as study treatment at that time. Patients who prefer the treatment that is not offered as study treatment in that particular centre at that time (e.g. active surveillance in a centre that has not yet crossed over into the active surveillance group) cannot be included in the trial. These patients will be offered their preferred treatment in the same centre outside the trial. For both treatment arms, separate patient information letters are available. Eligible patients will receive one of the two patient information letters, depending on the study treatment in a particular centre at the time of recruitment. A period of at least a week will be given to subjects to consider their decision. Both patient information letters and informed consent forms are attached as separate documents.

11.3 Objection by minors or incapacitated subjects NA

11.4 Benefits and risks assessment, group relatedness

The main burden for participating patients is to undergo one or more rounds of additional diagnostic tests (clinical response evaluations, CRE) after completion of neoadjuvant chemoradiotherapy. The number of rounds will depend on whether residual disease is detected during the CREs. These CREs will consist of endoscopy, endoscopic ultrasonography (EUS) +/- fine needle aspiration (FNA) and PET-CT. All three tests carry a minimal risk of complications. Preliminary results from the preSANO-trial (after 140 patients who completed response evaluations) show only one mucosal tear during endoscopy, which did not have any clinical consequences, and one cardiac arrhythmia after endoscopy, unrelated to the endoscopy and without clinical consequences. FNA was safely performed in 33 patients.
The main risk for participating patients is that tumour regrowths might be detected beyond the resectability limit, that distant metastases develop from local regrowths and that delayed surgery is potentially associated with an increase in postoperative morbidity. Therefore, an intensive surveillance regimen has been incorporated and strict stopping rules have been formulated (see §8.7). Consequently, patients in the active surveillance arm will undergo up to 10 additional PET-CT-scans during participation in the trial. The total radiation dose of 10 PET-CT-scans for an average patient will be approximately 64 mSv. Based on the radiation dose calculations, the risk of total detriment associated with the additional radiation exposure is greater than 1 in 1000 and the risk assessment for the SANO trial is therefore category III. However, this

radiation dose is about 1/1200 of the radiation dose that is applied during nCRT according to the CROSS regimen. Moreover, the majority of patients in the active surveillance arm (+/- 70%) will avoid oesophagectomy and the associated risks (e.g. postoperative mortality +/- 4%). Furthermore, it is expected that the majority of included patients is above 60 years of age, thereby biologically reducing the long-term detriment associated with the additional radiation exposure. Taken together, the exposure to radiation resulting from additional PET-CT-scans during the trial is justified, since it is expected that the benefit of active surveillance substantially outweighs the possible risks of additional radiation exposure. Participating patients may benefit by avoiding an oesophagectomy, which is associated with severe postoperative morbidity, relatively high postoperative mortality and a substantial impact on patients' quality of life. Moreover, it is anticipated that ultimately health care costs will substantially decrease.

11.5 Compensation for injury

The sponsor/investigator has a liability insurance which is in accordance with article 7, subsection 6 of the WMO.

The sponsor (also) has an insurance which is in accordance with the legal requirements in the Netherlands (Article 7 WMO and the Measure regarding Compulsory Insurance for Clinical Research in Humans of 23th June 2003). This insurance provides cover for damage to research subjects through injury or death caused by the study.

1. € 650.000,-- (i.e. six hundred and fifty thousand Euro) for death or injury for each subject who participates in the Research;
2. € 5.000.000,-- (i.e. five million Euro) for death or injury for all subjects who participate in the Research;
3. € 7.500.000,-- (i.e. seven million five hundred thousand Euro) for the total damage incurred by the organisation for all damage disclosed by scientific research for the Sponsor as 'verrichter' in the meaning of said Act in each year of insurance coverage.

The insurance applies to the damage that becomes apparent during the study or within 4 years after the end of the study.

12. ADMINISTRATIVE ASPECTS, MONITORING AND PUBLICATION

12.1 Handling and storage of data and documents

Data will be handled confidentially and anonymously. Upon inclusion into this study, each patient will be assigned a study number. This study number will be listed on all study related documentation. In this study, no personal documents will be listed. The key to the code will be stored in a separate document. When it is necessary to trace data to an individual subject, a subject identification code list will be used to link the data to the subject. The code will not be based on the patient's initials and birth-date. The key to the code will be accessible only by the principal investigator, coordinating investigator and datamanager.
The handling of personal data is in compliance with the Dutch Personal Data Protection Act (in Dutch: De Wet Bescherming Persoonsgegevens, Wbp).

To enhance uniformity of data collection, the sponsor intends to outsource the Local Datamanagement for all participating sites to the Clinical Trial Center (CTC) of the Erasmus MC. The CTC will thus be responsible for data collection and completion of the eCRF for all included patients in all participating sites. For reasons of efficiency, participating sites will be requested to provide the CTC Local Datamanager a Remote Access account, granting the CTC Local Datamanager access to the trial subjects' patient dossier remotely (i.e. from the secure Erasmus MC environment). The CTC will abide by and take into account the site's Local Policy and current legal and regulatory frameworks

12.2 Monitoring and Quality Assurance

A study specific monitoring plan, compliant with NFU guidelines and the Erasmus MC requirements for the METC approved monitoring risk (i.e. minimal), will be written.

12.3 Amendments

Amendments are changes made to the research after a favourable opinion by the accredited METC has been given. All amendments will be notified to the METC that gave a favourable opinion.

A 'substantial amendment' is defined as an amendment to the terms of the METC application, or to the protocol or any other supporting documentation, that is likely to affect to a significant degree:

- the safety or physical or mental integrity of the subjects of the trial;
- the scientific value of the trial;
- the conduct or management of the trial; or
- the quality or safety of any intervention used in the trial.

All substantial amendments will be notified to the METC and to the competent authority.

Non-substantial amendments will not be notified to the accredited METC and the competent authority, but will be recorded and filed by the sponsor.

12.4 Annual progress report

The sponsor/investigator will submit a summary of the progress of the trial to the accredited METC once a year. Information will be provided on the date of inclusion of the first subject, numbers of subjects included and numbers of subjects that have completed the trial, serious adverse events and serious adverse reactions, other problems, and amendments.

12.5 End of study report

The investigator will notify the accredited METC of the end of the study within a period of 8 weeks. The end of the study is defined as the last patient's last visit.

The sponsor will notify the METC immediately of a temporary halt of the study, including the reason of such an action.

In case the study is ended prematurely, the sponsor will notify the accredited METC within 15 days, including the reasons for the premature termination.

Within one year after the end of the study, the investigator/sponsor will submit a final study report with the results of the study, including any publications/abstracts of the study, to the accredited METC.

12.6 Public disclosure and publication policy

The protocol and the (final) results of the study will be summarized in a report / article and will be submitted for publication in a medical journal. Also, all participating patients or their family will receive a layman's summary of the (final) results of the study.

This clinical trial will be registered in the Netherlands Trial Register.

13. STRUCTURED RISK ANALYSIS NA

<This chapter is applicable for research with any product: medicinal product, food product, medical device or other (as described in chapter 6 and 7)>

13.1 Potential issues of concern NA

a. Level of knowledge about mechanism of action

b. Previous exposure of human beings with the test product(s) and/or products with a similar biological mechanism

c. Can the primary or secondary mechanism be induced in animals and/or in *ex-vivo* human cell material?

d. Selectivity of the mechanism to target tissue in animals and/or human beings

e. Analysis of potential effect

f. Pharmacokinetic considerations

g. Study population

h. Interaction with other products

i. Predictability of effect

j. Can effects be managed?

13.2 Synthesis NA

14. APPENDICES

Appendix A 7th TNM-Staging System (incl. lymph node stations)

Primary tumor (T)*

TX Primary tumor cannot be assessed
T0 No evidence of primary tumor
Tis High-grade dysplasia•
T1 Tumor invades lamina propria, muscularis mucosae, or submucosa
T1a Tumor invades lamina propria or muscularis mucosae
T1b Tumor invades submucosa
T2 Tumor invades muscularis propria
T3 Tumor invades adventitia
T4 Tumor invades adjacent structures
T4a Resectable tumor invading pleura, pericardium, or diaphragm
T4b Unresectable tumor invading other adjacent structures, such as aorta, vertebral body, trachea, etc.

Regional lymph nodes (N)∆

NX Regional lymph node(s) cannot be assessed
N0 No regional lymph node metastasis
N1 Metastasis in 1-2 regional lymph nodes
N2 Metastasis in 3-6 regional lymph nodes
N3 Metastasis in seven or more regional lymph nodes

Distant metastasis (M)

M0 No distant metastasis
M1 Distant metastasis

Histologic grade (G)

GX Grade cannot be assessed - stage grouping as G1
G1 Well differentiated
G2 Moderately differentiated
G3 Poorly differentiated
G4 Undifferentiated - stage grouping as G3 squamous

**Stage**	**T**	**N**	**M**	**Grade**	**Tumor location §**
**0**	Tis (HGD)	N0	M0	1, X	Any
**IA**	T1	N0	M0	1, X	Any
**IB**	T1	N0	M0	2-3	Any
	T2-3	N0	M0	1, X	Lower, X
**IIA**	T2-3	N0	M0	1, X	Upper, middle
	T2-3	N0	M0	2-3	Lower, X
**IIB**	T2-3	N0	M0	2-3	Upper, middle
	T1-2	N1	M0	Any	Any
	T1-2	N2	M0	Any	Any
**IIIA**	T3	N1	M0	Any	Any
	T4a	N0	M0	Any	Any
**IIIB**	T3	N2	M0	Any	Any
	T4a	N1-2	M0	Any	Any
**IIIC**	T4b	Any	M0	Any	Any
	Any	N3	M0	Any	Any
**IV**	Any	Any	M1	Any	Any

Note: cTNM is the clinical classification, pTNM is the pathologic classification.
* At least maximal dimension of the tumor must be recorded and multiple tumours require the T(m) suffix.
• High-grade dysplasia (HGD) includes all non-invasive neoplastic epithelia that was formerly called carcinoma in situ, a diagnosis that is no longer used for columnar mucosae anywhere in the gastrointestinal tract.
∆ Number must be recorded for total number of regional nodes sampled and total number of reported nodes with metastasis.
◊ Or mixed histology including a squamous component or NOS.
§ Location of the primary cancer site is defined by the position of the upper (proximal) edge of the tumor in the oesophagus.
Used with the permission of the American Joint Committee on Cancer (AJCC), Chicago, Illinois. The original source for this material is the AJCC Cancer Staging Manual, Seventh Edition (2010) published by Springer New York, Inc.

Map taken from Rice Chest Surg Clin N Am (61, 62)

anterior view

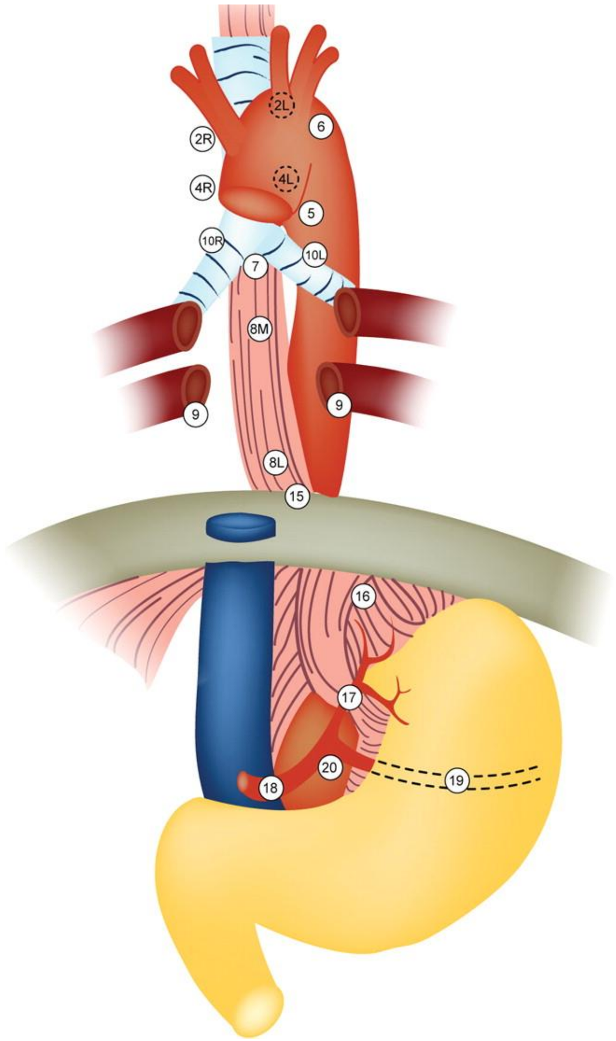

left lateral view

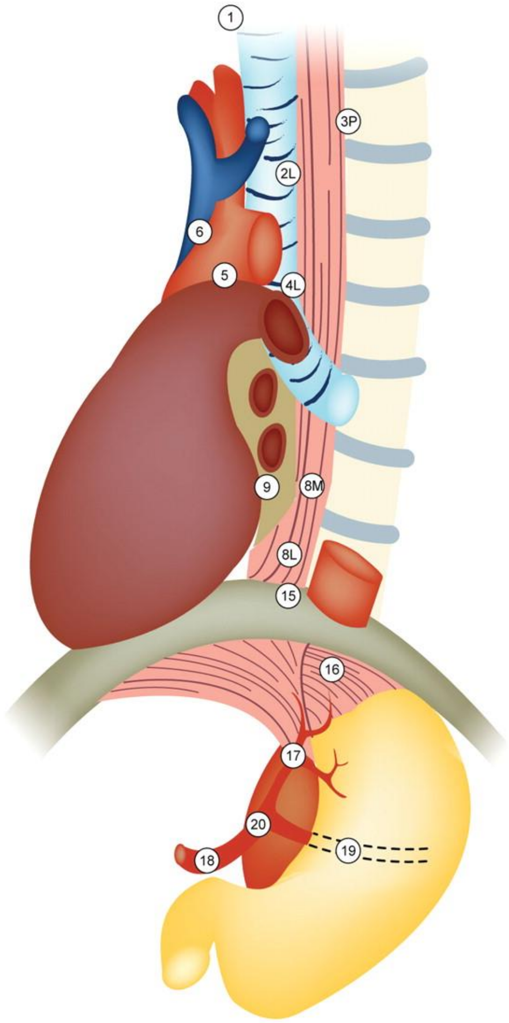

Lymph node stations:

1 = supraclavicular,
2L = left paratracheal
2R = right paratracheal
3P = posterior mediastinal
4L = left tracheobronchial angle
4R = right tracheobronchial angle
5 = aortopulmonary = anterior mediastinal
7 = subcarinal
8L = lower para-oesophageal
8M = middle para-oesophageal
9 = inferior pulmonary ligament
10L = left hilar
10R = right hilar
15 = diaphragmatic
16 = paracardial
17 = left gastric
18 = common hepatic
19 = splenic
20 = celiac

Appendix B Contact persons of the SANO study group (provisional)

Coordinating Investigator		
Drs. B.J. van der Wilk	Surgery	Erasmus MC
Drs. B. Eyck	Surgery	Erasmus MC
Project Leader		
Prof. dr. J.J.B. van Lanschot	Surgery	Erasmus MC
Principal Investigators		
Dr. S.M. Lagarde	Surgery	Erasmus MC
Dr. B.P.L. Wijnhoven	Surgery	Erasmus MC
Erasmus MC, Rotterdam		
Dr. K. Biermann	Pathology	Erasmus MC
Dr. A. van der Gaast	Medical Oncology	Erasmus MC
Dr. W.G. Ista	Implementation Fellow	Erasmus MC
Dr. N.C. Krak	Radiology	Erasmus MC
Dr. J.J.M.E. Nuyttens	Radiotherapy	Erasmus MC
Dr. S. Polinder	Health Economics	Erasmus MC
Dr. M.C.W. Spaander	Gastroenterology	Erasmus MC
Prof. dr. E.W. Steyerberg	Public Health	Erasmus MC
Dr. R. Valkema	Nuclear Medicine	Erasmus MC
Almelo		
Dr. A. Agool	Nuclear Medicine	Zorggroep Twente
Drs. J. van Baarlen	Pathology	Lab PON
Drs. E.M. Hendriksen	Radiotherapy	Medisch Spectrum Twente
Dr. R. Hoekstra	Medical Oncology	Zorggroep Twente
Dr. E.A. Kouwenhoven	Surgery	Zorggroep Twente
Drs. A. van der Linde	Gastroenterology	Zorggroep Twente
Amsterdam		
Dr. J. van Dieren	Medical Oncology/Gastroenterology	AVL-NKI
Dr. J. van Sandick	Surgery	AVL-NKI
Dr. P. Snaebjornsson	Pathology	AVL-NKI
Dr. E. Owens	Nuclear Medicine	AVL-NKI
Drs. F.E.M. Voncken	Radiotherapy	AVL-NKI
Apeldoorn		
Dr. H. Doornewaard	Pathology	Gelre Ziekenhuis
Drs. G.W. Erkelens	Gastroenterology	Gelre Ziekenhuis
Drs. D. de Koning	Gastroenterology	Gelre Ziekenhuis
Drs. S.C.S. Tromp – van Driel	Medical Oncology	Gelre Ziekenhuis
Dr. E.S van der Zaag	Surgery	Gelre Ziekenhuis
Drs. M.D. Zuijdwijk	Nuclear Medicine	Gelre Ziekenhuis
Dr. K Muller	Radiotherapy	Gelre Ziekenhuis
Delft		
Drs. M.R.J. ten Broek	Nuclear Medicine	Reinier de Graaf Group
Drs. R.J. Dallinga	Radiology	Reinier de Graaf Group
Dr. J.W.T. Dekker	Surgery	Reinier de Graaf Group
Dr. V.O. Dezentjé	Medical Oncology	Reinier de Graaf Group
Dr. R.R. de Krijger	Pathology	Reinier de Graaf Group
Dr. K.J. Neelis	Radiotherapy	Reinier de Graaf Group
Drs. R. Quispel	Gastroenterology	Reinier de Graaf Group
Eindhoven		
Dr. G.J. Creemers	Medical Oncology	Catharina Cancer Center, Eindhoven
Dr. G.A.P. Nieuwenhuijzen	Surgery	Catharina Cancer Center, Eindhoven
Dr. M.C. van der Sangen	Radiotherapy	Catharina Cancer Center, Eindhoven

Dr. E.J. Schoon	Gastroenterology	Catharina Cancer Center, Eindhoven
Dr. D.N.J. Wyndaele	Nuclear Medicine	Catharina Cancer Center, Eindhoven
Dr. G. van Lijnschoten	Pathology	PAMM
Heerlen		
Dr. J. Buijsen	Radiotherapy	Maastro Clinic
Dr. R.G. Riedl	Pathology	Zuyderland MC
Drs. W.M.J. Schreurs	Nuclear Medicine	Zuyderland MC
Dr. M.N. Sosef	Surgery	Zuyderland MC
Dr. L.E. Oostenbrug	Gastroenterology	Zuyderland MC
Drs. F.A.R.M. Warmerdam	Medical Oncology	Zuyderland MC
Leiden		
Dr. H.H. Hartgrink	Surgery	LUMC
Dr. J.J. Boonstra	Gastroenterology	LUMC
Dr. M. Slingerland	Medical Oncology	LUMC
Dr. I.M. Lips	Radiotherapy	LUMC
To be determined	Nuclear Medicine	
To be determined	Radiology	
To be determined	Pathology	
Leeuwarden		
Dr. H. Balink	Nuclear Medicine	Medisch Centrum Leeuwarden
Dr. W.E. Fiets	Medical Oncology	Medisch Centrum Leeuwarden
Dr. K. van der Linde	Gastroenterology	Medisch Centrum Leeuwarden
Dr. J. Nieken	Pathology	Medisch Centrum Leeuwarden
Drs. V. Oppedijk	Radiotherapy	Radiotherapeutisch Instituut Friesland
Prof. dr. J.P.E.N. Pierie	Surgery	Medisch Centrum Leeuwarden
Drs. R. Wolf	Radiology	Medisch Centrum Leeuwarden
Maasstad Ziekenhuis, Rotterdam		
Dr. P.P.L.O. Coene	Surgery	Maasstad Ziekenhuis
Dr. E.F. Courech Staal	Nuclear Medicine	Maasstad Ziekenhuis
Dr. M. Kliffen	Pathology	Maasstad Ziekenhuis
Dr. E.M.M. Kuiper	Gastroenterology	Maasstad Ziekenhuis
Drs. H.T. Teng	Radiology	Maasstad Ziekenhuis
Nijmegen		
Dr. M.J.R. Janssen	Nuclear Medicine	Radboud UMC
Drs. M.H. Liedenbaum	Radiology	Radboud UMC
Drs. C. van der Post	Pathology	Radboud UMC
Dr. S.A. Radema	Medical Oncology	Radboud UMC
Prof. dr. C. Rosman	Surgery	Radboud UMC
Drs. H. Rütten / Dr. P.M. Braam	Radiotherapy	Radboud UMC
Prof. dr. P.D. Siersema	Gastroenterology	Radboud UMC
Tilburg		
Dr. L.V. Beerepoot or	Medical Oncology	Elisabeth Tweesteden Ziekenhuis
Dr. G.H.A. Dodemont or	Gastroenterology	Elisabeth Tweesteden Ziekenhuis
Dr. J. Heisterkamp	Surgery	Elisabeth Tweesteden Ziekenhuis
Drs. J.C. van Oord	Radiology	Elisabeth Tweesteden Ziekenhuis
Drs. T. Rozema	Radiotherapy	Instituut Verbeeten
Dr. I.A.C. Vermeltfoort	Nuclear Medicine	Instituut Verbeeten
Dr. A.A.M. van der Wurff	Pathology	Elisabeth Tweesteden Ziekenhuis

Appendix C Richtlijnlijn auteurschappen (concept, december 2016)

Algemene overwegingen:

De meeste tijdschriften maken een onderscheid tussen:

a) *hoofdauteurs boven artikel:*

deze staan vermeld boven het artikel. Dit aantal is vaak gelimiteerd tot bijvoorbeeld 10 personen ("The British Journal of Surgery holds the view that in the context of surgical publishing most articles are unlikely to involve significant contributions from more than ten authors").

b) medeonderzoekers = 'collaborators' = *leden van de onderzoeksgroep onder artikel:*

alle leden van de onderzoeksgroep staan vermeld aan het einde van het artikel als 'collaborators'. Deze namen worden allen vermeld in PubMed. De bijdrage van de collaborators die niet boven het artikel vermeld staan verschilt inhoudelijk van de bijdrage van hen die wel boven het artikel vermeld staan. Ook deze medeonderzoekers moeten hun positie echter 'verdienen'. Het baart de Editorial Board van bijvoorbeeld Br J Surg zorgen dat in het decembernummer van 2014 een artikel is verschenen waarin 98 patiënten worden beschreven door 54 collaborators. Naar de mening van de editors moet de positie van iedere collaborator verdedigbaar en gefundeerd zijn volgens de richtlijnen van de *International Committee of Medical Journal Editors* (www.icmje.org). Vrijwel alle internationale tijdschriften committeren zich aan deze richtlijnen:

"The ICMJE recommends that authorship be based on the following 4 criteria:

- Substantial contributions to the conception or design of the work; or the acquisition, analysis, or interpretation of data for the work; AND
- Drafting the work or revising it critically for important intellectual content; AND
- Final approval of the version to be published; AND
- Agreement to be accountable for all aspects of the work in ensuring that questions related to the accuracy or integrity of any part of the work are appropriately investigated and resolved."

> "All those designated as authors should meet all four criteria for authorship, and all who meet the four criteria should be identified as authors. Those who do not meet all four criteria should be acknowledged. These authorship criteria are intended to reserve the status of authorship for those who deserve credit and can take responsibility for the work. The criteria are not intended for use as a means to disqualify colleagues from authorship who otherwise meet authorship criteria by denying them the opportunity to meet criterion #1, 2 or 3. Therefore, all individuals who meet the first criterion should have the opportunity to participate in the review, drafting, and final approval of the manuscript."

c) *personen in acknowledgements:*

personen die een bijdrage hebben geleverd aan de totstandkoming van het artikel, maar die niet kwalificeren als medeonderzoekers kunnen vermeld worden aan het einde van het artikel onder 'acknowledgements'. Dit kunnen datamanagers zijn, maar bijvoorbeeld ook geïnterviewde collegae. In de Erasmus MC 'guidelines on authorship' staat over dit onderwerp o.a. het volgende vermeld: "A co-authorship is not justified by the routine provision of data or material, or by ensuring the necessary funding. Sufficient acknowledgement of such contributions can be provided by a mention in the 'acknowledgements' or in an overview of those who have contributed."

Bij multidisciplinaire onderzoeken die in meerdere ziekenhuizen worden uitgevoerd moet in redelijkheid worden afgesproken hoe met het grote aantal potentiële co-auteurs wordt omgegaan. Bij de SANO trial zijn in elk van de (10-) 12 participerende ziekenhuizen zeven afdelingen in wisselende mate betrokken, te weten:

a) Heelkunde
b) MDL
c) Nucleaire Geneeskunde
d) Pathologie
e) Radiologie
f) Medische Oncologie
g) Radiotherapie

Inclusief de twee principal investigators, de beide arts-onderzoekers/promovendi en de methodoloog/statisticus zijn er dus tenminste 100 personen, die potentieel kwalificeren als hoofdauteur dan wel collaborator binnen de SANO trial waarbij in totaal 600 patiënten zullen worden geïncludeerd waarvan 300 patiënten (d.w.z. 1 auteur/collaborator per 3 patiënten). Enige prudentie lijkt daarmee op zijn plaats.

<u>**Concept-voorstel voor auteurschappen**</u>:

Op grond van bovenstaande argumenten komen wij tot het volgende voorstel:

1. ongeacht de uiteindelijke inclusie mag elke participerende afdeling van elk participerend centrum één lid van de ‘SANO-study group’ benoemen. Alle leden van deze onderzoeksgroep worden tezamen vermeld aan het einde van het manuscript als co-auteurs/medeonderzoekers.

2. ongeacht de uiteindelijke inclusie mag elk participerend centrum zelf uit zijn midden één lid van de onderzoeksgroep aanwijzen die optreedt als hoofdauteur die boven het artikel vermeld wordt.

3. daarnaast treden de twee principal investigators, de beide arts-onderzoekers en de methodoloog/ statisticus uit het coördinerende centrum op als leden van de onderzoeksgroep en als hoofdauteurs.

4. een participerend centrum dat tenminste 5% van de patiënten includeert (≥ 30 patiënten), mag naar eigen keuze één extra lid aanwijzen van de onderzoeksgroep en bovendien uit de onderzoeksgroep nog één extra hoofdauteur aanwijzen.

5. een participerend centrum dat tenminste 10% van de patiënten includeert (≥ 60 patiënten), mag naar keuze nog één extra lid van de onderzoeksgroep aanwijzen en bovendien uit de onderzoeksgroep nog één extra hoofdauteur aanwijzen.

6. een participerend centrum dat tenminste 15% van de patiënten includeert (≥ 90 patiënten), mag naar keuze nog één extra lid van de onderzoeksgroep aanwijzen en bovendien uit de onderzoeksgroep nog één extra hoofdauteur aanwijzen …….. etc.

7. op deze wijze eindigen wij waarschijnlijk in totaal met 100-110 leden van de onderzoeksgroep en 35 hoofdauteurs.

8. het staat elk participerend centrum vrij om met een gezamenlijk voorstel te komen om binnen het aan dit centrum toegekende aantal hoofdauteurs en leden van de onderzoeksgroep verschuivingen in de vertegenwoordigers van de participerende afdelingen aan te brengen, als daartoe op grond van de mate van betrokkenheid aanleiding bestaat.

9. naar verwachting zullen meerdere publicaties voortkomen uit de SANO trial. Bovengenoemde afspraken blijven dan gehandhaafd, maar afhankelijk van de wetenschappelijke focus van een manuscript kunnen de vertegenwoordigende hoofdauteurs uit de verschillende centra variëren (meer nucleair-geneeskundigen als hoofdauteur bij een nucleair-geneeskundig onderwerp, meer pathologen als hoofdauteur bij een pathologie onderwerp etc.)

10. ten aanzien van de volgorde van de hoofdauteurs worden de volgende uitgangspunten gehanteerd, waarvan beargumenteerd kan worden afgeweken:

- eerste: de meest voor de hand liggende promovendus
- tweede: vertegenwoordiger uit 1^{e} = grootste centrum
- derde: 1^{e} principal investigator

- vierde: vertegenwoordiger uit 2^{e} = een na grootste centrum
- vijfde: de andere promovendus
- zesde: 2^{e} principal investigator
- zevende en volgenden : in alfabetische volgorde

- een-na-laatste: methodoloog/statisticus
- laatste: project leader.

11. alle hoofdauteurs dienen daarnaast te voldoen aan de richtlijnen publiceren en auteurschappen van het Erasmus MC.(63)

Rotterdam, december 2016.

Bas Wijnhoven, Manon Spaander en Jan van Lanschot.

(richtlijn auteurschappen publicaties SANO resultaten(1).docx)

Appendix D Amendment for MR side study (MR-SANO; Antoni van Leeuwenhoek, only for patients in Antoni van Leeuwenhoek)

Study amendment May 11th, 2017:

Aim of the study

To explore the value of magnetic resonance imaging (MRI) for response prediction before and during neoadjuvant chemoradiotherapy (nCRT) and response assessment after nCRT in patients with oesophageal cancer and investigate its value in detection of residual disease or local regrowth for patients with active surveillance policy.

Background

The diagnostic modalities nowadays used for oesophageal tumour staging are computed tomography (CT), 18-fluorodeoxyglucose positron emission tomography (FDG-PET), (echo-)endoscopy. Currently state of the art MRI is emerging as an additional imaging technique in oesophageal cancer. When locoregional MR images are derived with navigation-guidance, MR images of the oesophageal tumour show good image quality (64). Diffusion-weighted MRI (DW-MRI) is promising in response prediction; it seems to be of diagnostic value for prediction of tumour aggressiveness when assessed before treatment and as predictor of histopathologic response when assessed during the first 2-3 weeks of nCRT (65-67).

Description

The MR-SANO study combines the SANO study with additional locoregional MRI of the oesophagus for response prediction, response assessment and detection of local regrowth of patients diagnosed with oesophageal carcinoma. Therefore, all included patients undergo locoregional MRI at the clinical response evaluation moments as described in the SANO protocol. MRI will be performed at time of diagnosis, after 2 weeks of neoadjuvant chemoradiotherapy (nCRT), during clinical response evaluation moments (CRE-I and CRE-II) and at follow-up moments of patients in the surveillance arm, in addition to the investigational procedures of the SANO protocol. For evaluation moments where endoscopy is included, the MRI is planned prior to the endoscopy, to enable the results of the MRI to be incorporated during endoscopy. The results of the MRI are discussed before endoscopy by an experienced radiologist and the gastro-enterologist performing the endoscopy. Subsequently, bite-on-bite forceps biopsies and/or fine needle aspirations are taken during endoscopy and/or EUS and will be based on the clinical finding of PET and endoscopy, combined with the MR-defined suspected areas for residual tumour and/or suspected lymph nodes.

In case of suspicion of residual disease or regrowth on MR imaging but not on PET and without pathological confirmation, the patient will not undergo surgery based on MR results only, but will continue surveillance. If the suspicion remains during follow-up, images will be submitted for review by the centralised multidisciplinary tumour board of the SANO study group.

Ad 3. Study Design

3.3 Study Overview

Pre-treatment work-up includes 18F-FDG PET-CT, endoscopy, EUS and locoregional focused MRI. MR imaging will also be acquired after 14 days of radiotherapy. At CRE-I and CRE-II, MRI of the primary tumour of the oesophagus

before endoscopy and EUS is assessed. In the active surveillance arm, MRI will be added to the predefined evaluation moments during follow-up.

3.4. Schedule of assessments*

The MR-SANO adds MRI in the schedule.

Parameter	Pretreatment	Neoadjuvant chemoradiotherapy (CROSS)	CRE-I	CRE-II (3 months after end of neoadjuvant chemoradio-therapy)	Active surveillance evaluations (6, 9, 12, 16, 20, 24, 30, 36, 48, 60 months after completion of neoadjuvant chemoradiotherapy[15])
**Eligibility check**	X				
**Written Informed consent**	X[10]				
**Inclusion**	X				
**"Randomisation" (treatment allocation)**				X[14]	
**Medical History**	X	X	X	X	X
**Physical Exam**	X	X	X	X	X
**ECOG Performance status** (Appendix B)	X	X	X	X	X
**Haematology**[1]	X	X			
**eGFR**	X	X			
**Biochemistry**[2]	X	X			
**Endoscopy + (random) bite-on-bite biopsies**	X		X	X	X
**Radial EUS**[3]	X			X	X
**Linear EUS (+FNA)**[4]	X			X	X
**CT of neck, thorax, abdomen and pelvis**	X				
**PET-CT (whole-body)**[19]	X	Optional[17]	X[8]	X[9]	X[9]
**MRI**[18]	X	X	X	X	X
**Pulmonary function tests**[5]	X				
**Bronchoscopy**[6]	X				
**ECG**	X				

Parameter	Pretreatment	Neoadjuvant chemoradiotherapy (CROSS)	CRE-I	CRE-II (3 months after end of neoadjuvant chemoradio-therapy)	Active surveillance evaluations (6, 9, 12, 16, 20, 24, 30, 36, 48, 60 months after completion of neoadjuvant chemoradiotherapy[15])
**Toxicity**[7]	Baseline	X			
**Quality of Life (EQ-5D, QLQ-C30, QLC-OG25 and Cancer Worry Scale)**	X			X	X[16]
**Surgery**			X[11]	X[12]	X[13]
**Postoperative complications**				X[12]	X[13]
**Pathology of resection specimen**				X[12]	X[13]

[1] Haematology: CBC, differential
[2] Biochemistry: serum protein, albumin, magnesium, electrolytes, serum creatinin, bilirubin, alkaline phosphatase, AST
[3] Radial EUS: with measurement of maximum tumour thickness and –area
[4] Linear EUS: with fine-needle aspiration (FNA) of any suspected lymph nodes
[5] Pulmonary function test: only on indication
[6] Bronchoscopy: when tumour is located above the carina and when there is suspicion for invasion of the tracheo-bronchial tree
[7] Toxicity: to be evaluated after each cycle
[8] PET-CT: during CRE-I, after OGD and EUS, only for non-complete clinical responders, to exclude disseminated disease
[9] PET-CT: during CRE-II and surveillance examinations, prior to OGD and EUS, for all patients (all were complete clinical responders during CRE-I) to guide OGD and EUS in targeting suspected locoregional laesions and to exclude disseminated disease
[10] Before inclusion, i.e. before **any** trial related procedure commences
[11] Only for patients with locoregional disease
[12] After CRE-II: Only for patients with cCR who are allocated to surgery
[13] Only for patients in whom a locoregional regrowth is highly suspected or proven, without any signs of distant dissemination
[14] After CRE-II: Only for patients with cCR
[15] Or when symptoms or results of any diagnostic test require shorter assessment intervals
[16] Quality of life will be assessed during the first 2 years only. Patients will be offered the possibility to summarize the quality of life outcomes in a logbook provided in the outpatient clinic. Patients will be asked to bring their personal logbook to the control appointments with the surgeon.
[17] An extra PET-CT will be acquired during neoadjuvant chemoradiotherapy in those patients (in AvL) who will also be participating in the MRI response project (Koningin Wilhelmina Fonds/Alpe research project number 10291) and will be used for research purpose only.
[18] MRI: during neoadjuvant chemoradiotherapy, CRE-I, CRE-II and response evaluations prior to OGD and EUS, for all patients to guide OGD and EUS.
[19] For patients allocated to surgery, a PET-CT will be performed at 12 and 24 months after completion of nCRT.

Ad 5. Treatment of subjects

MR-SANO side study adds a paragraph.

5.1.3. Magnetic resonance imaging

Patients will be scanned by MRI pre-treatment, during neoadjuvant chemoradiation, at CRE-I, CRE-II and during follow-up evaluation moments for active surveillance patients. The MRI exams will include a combination of anatomical and functional MRI scans. MRI will be evaluated by volumetric anatomical changes. Oesophagogastroscopy and endosonography takes place after MRI and biopsies/FNA will be taken from the area suspicious for residual tumour and/or suspected nodes.

Ad 8. Methods

MR-SANO side study adds diffusion-weighted MRI to these clinical response evaluations, as described above.

15. REFERENCES

1. Jemal A, Siegel R, Ward E, Hao Y, Xu J, Thun MJ. Cancer Statistics, 2009. CA: A Cancer Journal for Clinicians. 2009;59(4):225-49.
2. Hoeymans N MJ, Schoemaker CG (red.). . Gezondheid en determinanten. Deelrapport van de Volksgezondheid Toekomst Verkenning 2010 Van gezond naar beter. . Bilthoven: RIVM, 2010 Contract No.: RIVM-rapport nr. 270061006.
3. Gignoux M, Roussel A, Paillot B, Gillet M, Schlag P, Favre J-P, et al. The value of preoperative radiotherapy in esophageal cancer: Results of a study of the E.O.R.T.C. World Journal of Surgery. 1987;11(4):426-32.
4. Wang M, Gu XZ, Yin WB, Huang GJ, Wang LJ, Zhang DW. Randomized clinical trial on the combination of preoperative irradiation and surgery in the treatment of esophageal carcinoma: report on 206 patients. Int J Radiat Oncol Biol Phys. 1989;16(2):325-7.
5. Nygaard K, Hagen S, Hansen HS, Hatlevoll R, Hultborn R, Jakobsen A, et al. Pre-operative radiotherapy prolongs survival in operable esophageal carcinoma: A randomized, multicenter study of pre-operative radiotherapy and chemotherapy. The second scandinavian trial in esophageal cancer. World Journal of Surgery. 1992;16(6):1104-9.
6. Arnott SJ, Duncan W, Kerr GR, Walbaum PR, Cameron E, Jack WJ, et al. Low dose preoperative radiotherapy for carcinoma of the oesophagus: results of a randomized clinical trial. Radiother Oncol. 1992;24(2):108-13.
7. Kelsen DP, Ilson DH. Chemotherapy and combined-modality therapy for esophageal cancer. Chest. 1995;107(6 Suppl):224S-32S.
8. Seely AJE, Ivanovic J, Threader J, Al-Hussaini A, Al-Shehab D, Ramsay T, et al. Systematic classification of morbidity and mortality after thoracic surgery. Ann Thorac Surg. 2010;90(20732521):936-42.
9. Bailey SH, Bull DA, Harpole DH, Rentz JJ, Neumayer LA, Pappas TN, et al. Outcomes after esophagectomy: a ten-year prospective cohort. Ann Thorac Surg. 2003;75(12537219):217-22.
10. Donohoe CL, McGillycuddy E, Reynolds JV. Long-term health-related quality of life for disease-free esophageal cancer patients. World J Surg. 2011;35(8):1853-60.
11. Djarv T, Lagergren J, Blazeby JM, Lagergren P. Long-term health-related quality of life following surgery for oesophageal cancer. Br J Surg. 2008;95(18581441):1121-6.
12. Djarv T, Blazeby JM, Lagergren P. Predictors of postoperative quality of life after esophagectomy for cancer. J Clin Oncol. 2009;27(19289614):1963-8.
13. Verhoef C, van de Weyer R, Schaapveld M, Bastiaannet E, Plukker JT. Better survival in patients with esophageal cancer after surgical treatment in university hospitals: a plea for performance by surgical oncologists. Ann Surg Oncol. 2007;14(5):1678-87.
14. Cooper JS, Guo MD, Herskovic A, Macdonald JS, Martenson JA, Jr., Al-Sarraf M, et al. Chemoradiotherapy of locally advanced esophageal cancer: long-term follow-up of a prospective randomized trial (RTOG 85-01). Radiation Therapy Oncology Group. JAMA. 1999;281(17):1623-7.
15. Allum WH, Stenning SP, Bancewicz J, Clark PI, Langley RE. Long-term results of a randomized trial of surgery with or without preoperative chemotherapy in esophageal cancer. J Clin Oncol. 2009;27(30):5062-7.
16. Ychou M, Boige V, Pignon JP, Conroy T, Bouche O, Lebreton G, et al. Perioperative chemotherapy compared with surgery alone for resectable gastroesophageal adenocarcinoma: an FNCLCC and FFCD multicenter phase III trial. J Clin Oncol. 2011;29(13):1715-21.
17. Sjoquist KM, Burmeister BH, Smithers BM, Zalcberg JR, Simes RJ, Barbour A, et al. Survival after neoadjuvant chemotherapy or chemoradiotherapy for resectable oesophageal carcinoma: an updated meta-analysis. Lancet Oncol. 2011;12(7):681-92.
18. van Hagen P, Hulshof MC, van Lanschot JJ, Steyerberg EW, van Berge Henegouwen MI, Wijnhoven BP, et al. Preoperative chemoradiotherapy for esophageal or junctional cancer. N Engl J Med. 2012;366(22):2074-84.

19. Shapiro J, van Lanschot JJ, Hulshof MC, van Hagen P, van Berge Henegouwen MI, Wijnhoven BP, et al. Neoadjuvant chemoradiotherapy plus surgery versus surgery alone for oesophageal or junctional cancer (CROSS): long-term results of a randomised controlled trial. Lancet Oncol. 2015;16(9):1090-8.
20. Shiozaki H, Sudo K, Xiao L, Wadhwa R, Elimova E, Hofstetter WL, et al. Distribution and timing of distant metastasis after local therapy in a large cohort of patients with esophageal and esophagogastric junction cancer. Oncology. 2014;86(5-6):336-9.
21. Habr-Gama A, Perez RO, Nadalin W, Sabbaga J, Ribeiro U, Jr., Silva e Sousa AH, Jr., et al. Operative versus nonoperative treatment for stage 0 distal rectal cancer following chemoradiation therapy: long-term results. Ann Surg. 2004;240(4):711-7; discussion 7-8.
22. Renehan AG, Malcomson L, Emsley R, Gollins S, Maw A, Myint AS, et al. Watch-and-wait approach versus surgical resection after chemoradiotherapy for patients with rectal cancer (the OnCoRe project): a propensity-score matched cohort analysis. Lancet Oncol. 2016;17(2):174-83.
23. Maas M, Beets-Tan RG, Lambregts DM, Lammering G, Nelemans PJ, Engelen SM, et al. Wait-and-see policy for clinical complete responders after chemoradiation for rectal cancer. J Clin Oncol. 2011;29(35):4633-40.
24. Mehanna H, Wong WL, McConkey CC, Rahman JK, Robinson M, Hartley AG, et al. PET-CT Surveillance versus Neck Dissection in Advanced Head and Neck Cancer. N Engl J Med. 2016;374(15):1444-54.
25. Allum WH, Stenning SP, Bancewicz J, Clark PI, Langley RE. Long-Term Results of a Randomized Trial of Surgery With or Without Preoperative Chemotherapy in Esophageal Cancer. Journal of Clinical Oncology. 2009;27(30):5062-7.
26. Oppedijk V, Van Der Gaast A, Van Lanschot JJB, Van Hagen P, Van Os R, Van Rij CM, et al. Patterns of recurrence after surgery alone versus preoperative chemoradiotherapy and surgery in the CROSS trials. Journal of Clinical Oncology. 2014;32(5):385-91.
27. Chambers AF, Groom AC, MacDonald IC. Metastasis: Dissemination and growth of cancer cells in metastatic sites. Nat Rev Cancer. 2002;2(8):563-72.
28. Versteijne E, van Laarhoven HW, van Hooft JE, van Os RM, Geijsen ED, van Berge Henegouwen MI, et al. Definitive chemoradiation for patients with inoperable and/or unresectable esophageal cancer: locoregional recurrence pattern. Dis Esophagus. 2015;28(5):453-9.
29. Sarkaria IS, Rizk NP, Bains MS, Tang LH, Ilson DH, Minsky BI, et al. Post-treatment endoscopic biopsy is a poor-predictor of pathologic response in patients undergoing chemoradiation therapy for esophageal cancer. Ann Surg. 2009;249(5):764-7.
30. Walsh TN, Bass GA, Furlong H, Kharytaniuk N, Mulsow JJW, Daly A, et al. Clinical complete response to chemo-radiotherapy in esophageal carcinoma: is surgery still required? Submitted for publication. 2015.
31. Shapiro J, ten Kate FJ, van Hagen P, Biermann K, Wijnhoven BP, van Lanschot JJ. Residual esophageal cancer after neoadjuvant chemoradiotherapy frequently involves the mucosa and submucosa. Ann Surg. 2013;258(5):678-88; discussion 88-9.
32. Swisher SG, Maish M, Erasmus JJ, Correa AM, Ajani JA, Bresalier R, et al. Utility of PET, CT, and EUS to identify pathologic responders in esophageal cancer. Ann Thorac Surg. 2004;78(4):1152-60; discussion -60.
33. Kim MK, Ryu JS, Kim SB, Ahn JH, Kim SY, Park SI, et al. Value of complete metabolic response by (18)F-fluorodeoxyglucose-positron emission tomography in oesophageal cancer for prediction of pathologic response and survival after preoperative chemoradiotherapy. Eur J Cancer. 2007;43(9):1385-91.
34. Taketa T, Correa AM, Suzuki A, Blum MA, Chien P, Lee JH, et al. Outcome of trimodality-eligible esophagogastric cancer patients who declined surgery after preoperative chemoradiation. Oncology. 2012;83(5):300-4.
35. Taketa T, Xiao L, Sudo K, Suzuki A, Wadhwa R, Blum MA, et al. Propensity-based matching between esophagogastric cancer patients who had surgery and who declined surgery after preoperative chemoradiation. Oncology. 2013;85(2):95-9.
36. Castoro C, Scarpa M, Cagol M, Alfieri R, Ruol A, Cavallin F, et al. Complete clinical response after neoadjuvant chemoradiotherapy for squamous cell cancer of the thoracic oesophagus: is surgery always necessary? J Gastrointest Surg. 2013;17(8):1375-81.

37. Jost C, Binek J, Schuller JC, Bauerfeind P, Metzger U, Werth B, et al. Endosonographic radial tumor thickness after neoadjuvant chemoradiation therapy to predict response and survival in patients with locally advanced esophageal cancer: a prospective multicenter phase II study by the Swiss Group for Clinical Cancer Research (SAKK 75/02). Gastrointest Endosc. 2010;71(7):1114-21.
38. Noordman BJ, Spaander MCW, Valkema R, Wijnhoven BPL, van Berge Henegouwen MI, Shapiro J, et al. Detection of residual disease after neoadjuvant chemoradiotherapy for oesophageal cancer (preSANO): a prospective multicentre, diagnostic cohort study. Lancet Oncol. 2018;19(7):965-74.
39. Noordman BJ, Wijnhoven BP, van Lanschot JJ. Optimal surgical approach for esophageal cancer in the era of minimally invasive esophagectomy and neoadjuvant therapy. Dis Esophagus. 2015.
40. Shapiro J, van Hagen P, Lingsma HF, Wijnhoven BP, Biermann K, Ten Kate FJ, et al. Prolonged time to surgery after neoadjuvant chemoradiotherapy increases histopathological response without affecting survival in patients with esophageal or junctional cancer. Ann Surg. 2014;260(5):807-14.
41. Kim JY, Correa AM, Vaporciyan AA, Roth JA, Mehran RJ, Walsh GL, et al. Does the timing of esophagectomy after chemoradiation affect outcome? Ann Thorac Surg. 2012;93(1):207-12; discussion 12-3.
42. Ruol A, Rizzetto C, Castoro C, Cagol M, Alfieri R, Zanchettin G, et al. Interval between neoadjuvant chemoradiotherapy and surgery for squamous cell carcinoma of the thoracic esophagus: does delayed surgery have an impact on outcome? Ann Surg. 2010;252(5):788-96.
43. Kathiravetpillai N, Koeter M, van der Sangen MJ, Creemers GJ, Luyer MD, Rutten HJ, et al. Delaying surgery after neoadjuvant chemoradiotherapy does not significantly influence postoperative morbidity or oncological outcome in patients with oesophageal adenocarcinoma. Eur J Surg Oncol. 2016;42(8):1183-90.
44. Furlong H, Bass G, Breathnach O, O'Neill B, Leen E, Walsh TN. Targeting therapy for esophageal cancer in patients aged 70 and over. J Geriatr Oncol. 2013;4(2):107-13.
45. Blazeby JM, Strong S, Donovan JL, Wilson C, Hollingworth W, Crosby T, et al. Feasibility RCT of definitive chemoradiotherapy or chemotherapy and surgery for oesophageal squamous cell cancer. Br J Cancer. 2014;111(2):234-40.
46. Earlam R. An MRC prospective randomised trial of radiotherapy versus surgery for operable squamous cell carcinoma of the oesophagus. Ann R Coll Surg Engl. 1991;73(1):8-12.
47. Du CY, Zhou Y, Song C, Wang YP, Jie ZG, He YL, et al. Is there a role of surgery in patients with recurrent or metastatic gastrointestinal stromal tumours responding to imatinib: a prospective randomised trial in China. Eur J Cancer. 2014;50(10):1772-8.
48. Bonvalot S. Is there a role for surgery in patients with recurrent or metastatic gastrointestinal stromal tumours responding to imatinib: a prospective randomised trial. Personal communication.
49. Brown CA, Lilford RJ. The stepped wedge trial design: a systematic review. BMC Med Res Methodol. 2006;6:54.
50. Hemming K, Haines TP, Chilton PJ, Girling AJ, Lilford RJ. The stepped wedge cluster randomised trial: rationale, design, analysis, and reporting. BMJ. 2015;350:h391.
51. Baio G, Copas A, Ambler G, Hargreaves J, Beard E, Omar RZ. Sample size calculation for a stepped wedge trial. Trials. 2015;16:354.
52. van der Wilk BJ, Noordman BJ, Neijenhuis LKA, Nieboer D, Nieuwenhuijzen GAP, Sosef MN, et al. Active Surveillance Versus Immediate Surgery in Clinically Complete Responders After Neoadjuvant Chemoradiotherapy for Esophageal Cancer: A Multicenter Propensity Matched Study. Ann Surg. 2019.
53. Boellaard R, O'Doherty MJ, Weber WA, Mottaghy FM, Lonsdale MN, Stroobants SG, et al. FDG PET and PET/CT: EANM procedure guidelines for tumour PET imaging: version 1.0. Eur J Nucl Med Mol Imaging. 2010;37(1):181-200.
54. Low DE, Alderson D, Cecconello I, Chang AC, Darling GE, D'Journo XB, et al. International Consensus on Standardization of Data Collection for Complications Associated With

Esophagectomy: Esophagectomy Complications Consensus Group (ECCG). Ann Surg. 2015;262(2):286-94.
55. Janssen MF, Pickard AS, Golicki D, Gudex C, Niewada M, Scalone L, et al. Measurement properties of the EQ-5D-5L compared to the EQ-5D-3L across eight patient groups: a multi-country study. Qual Life Res. 2013;22(7):1717-27.
56. Aaronson NK, Ahmedzai S, Bergman B, Bullinger M, Cull A, Duez NJ, et al. The European Organization for Research and Treatment of Cancer QLQ-C30: a quality-of-life instrument for use in international clinical trials in oncology. J Natl Cancer Inst. 1993;85(5):365-76.
57. Lagergren P, Fayers P, Conroy T, Stein HJ, Sezer O, Hardwick R, et al. Clinical and psychometric validation of a questionnaire module, the EORTC QLQ-OG25, to assess health-related quality of life in patients with cancer of the oesophagus, the oesophago-gastric junction and the stomach. Eur J Cancer. 2007;43(14):2066-73.
58. Custers JA, van den Berg SW, van Laarhoven HW, Bleiker EM, Gielissen MF, Prins JB. The Cancer Worry Scale: detecting fear of recurrence in breast cancer survivors. Cancer Nurs. 2014;37(1):E44-50.
59. Juweid ME, Stroobants S, Hoekstra OS, Mottaghy FM, Dietlein M, Guermazi A, et al. Use of positron emission tomography for response assessment of lymphoma: consensus of the Imaging Subcommittee of International Harmonization Project in Lymphoma. J Clin Oncol. 2007;25(5):571-8.
60. Shapiro J, Biermann K, van Klaveren D, Offerhaus GJ, Ten Kate FJ, Meijer SL, et al. Prognostic Value of Pretreatment Pathological Tumor Extent in Patients Treated With Neoadjuvant Chemoradiotherapy Plus Surgery for Esophageal or Junctional Cancer. Ann Surg. 2016.
61. Rice TW. Clinical staging of esophageal carcinoma. CT, EUS, and PET. Chest Surg Clin N Am. 2000;10(3):471-85.
62. Kim TJ, Kim HY, Lee KW, Kim MS. Multimodality assessment of esophageal cancer: preoperative staging and monitoring of response to therapy. Radiographics. 2009;29(2):403-21.
63. Rotterdam EM-UMC. Research Codes. Guidelines on publishing and authorship2011. p. 50 - 5.
64. van Rossum PS, van Hillegersberg R, Lever FM, Lips IM, van Lier AL, Meijer GJ, et al. Imaging strategies in the management of oesophageal cancer: what's the role of MRI? European radiology. 2013;23(7):1753-65.
65. Giganti F, Salerno A, Ambrosi A, Chiari D, Orsenigo E, Esposito A, et al. Prognostic utility of diffusion-weighted MRI in oesophageal cancer: is apparent diffusion coefficient a potential marker of tumour aggressiveness? La Radiologia medica. 2016;121(3):173-80.
66. van Rossum PSN, van Lier ALHMW, Reerink O, Lagendijk JJW, Meijer GJ, van Vulpen M, et al. Diffusion-weighted magnetic resonance imaging for the prediction of pathologic response to neoadjuvant chemoradiotherapy in esophageal cancer. Radiotherapy and Oncology. 2015;115(2):163-70.
67. Wang L, Han C, Zhu S, Shi G, Wang Q, Tian H, et al. Investigation of using diffusion-weighted magnetic resonance imaging to evaluate the therapeutic effect of esophageal carcinoma treatment. Oncology research and treatment. 2014;37(3):112-6.
